# Antimicrobial resistance interventions in the animal sector: scoping review

**DOI:** 10.3389/frabi.2023.1233698

**Published:** 2023-08-31

**Authors:** Alice B. J. E. Jacobsen, Jane Ogden, Abel B. Ekiri

**Affiliations:** ^1^ Department of Comparative Biomedical Sciences, School of Veterinary Medicine, University of Surrey, Guildford, United Kingdom; ^2^ Department of Psychological Sciences, School of Psychology, University of Surrey, Guildford, United Kingdom

**Keywords:** antimicrobial resistance, interventions, antimicrobial stewardship, antimicrobial usage, behavioural science, One Health, animal

## Abstract

Animals are considered key contributors to the development and spread of antimicrobial resistance (AMR). However, little is known about the existing AMR interventions in the animal sector. This scoping review examines the existing evidence on AMR interventions aimed at livestock, animal health professionals (AHPs), and farmers, while reviewing their impact, limitations, gaps, and lessons for future use. The scoping review was conducted following guidelines from the PRISMA-ScR checklist. The databases, Web of Science, Scopus, PubMed, and international organisations’ websites (WHO, FAO, WOAH) were searched for articles reporting interventions targeting livestock, farmers, and AHPs. Interventions were categorised based on seven pre-defined primary measures including: change in antimicrobial use (AMU) practices; change in the uptake of antimicrobial stewardship (AMS); change in development of AMR; change in knowledge of appropriate AMU practices, AMR, and AMS; change in attitudes and perceptions concerning AMU, AMR, and AMS; and surveillance strategies. In total, ninety three sources were included: 66 studies, 20 reports, and 7 webpages. The reviewed interventions focused mostly on AMU practices (22/90), AMS uptake (8/90), and reduction of bacterial or resistant strains (30/90). Changes in knowledge (14/90) and attitude (1/90) were less frequently assessed and were often implicit. Most interventions were conducted within a select country (83/90) and 7/90 were at a global level. Only 19% (16/83) of interventions were implemented in low- and middle-income countries (LMICs) and most were at herd level with many self-reporting changes. Most of the interventions that focused on surveillance strategies (30/83) were implemented in high-income countries (62/83). Only one study investigated the financial implications of the intervention. The study findings provide an overview of existing AMR interventions and insights into the gaps which can be addressed to guide future interventions and research. A focus on developing, implementing and evaluating interventions in LMICs coupled with the use of objective outcome measures (e.g., measurable outcomes vs. self-reporting) will improve our understanding of the impact of interventions in these settings. Finally, assessing the financial benefits of interventions is necessary to inform feasibility and to encourage uptake of interventions aimed at reducing AMR in the animal health sector.

## 1 Introduction

Antimicrobial resistance (AMR) is a critical issue for both human and animal health (O’Neill, 2016). Globally, an estimated 1.27 million human deaths in 2019 were attributed to AMR (Murray et al., 2022). This is predicted to rise to 10 million in 2050 if no action is taken (O’Neill, 2016). AMR-attributed deaths in humans have been linked to the transfer of AMR- bacteria and AMR genes from animals to humans (Rhouma et al., 2022). The antimicrobials considered of high priority and essential for humans, Highest Priority Critically Important Antimicrobials (HPCIAs), are often used to treat resistant infections in animals (World Health Organization, 2018). The continued use of critically important antimicrobials (CIAs) in animals poses the risk of developing AMR, and onward transmission of CIA-resistant bacteria to humans which can reduce the effectiveness of the available CIAs (Tang et al., 2017). Other sources of AMR bacteria are community and hospital-acquired infections that can develop after misuse or overuse of antimicrobials in humans, lack of sanitation and diagnostics (e.g., lack of sensitivity testing and toilet/hand washing facility), and failure to use appropriate infection control measures in hospitals (World Health Organization, 2019). AMR infections can also be acquired from contaminated environments (World Health Organization, 2019; Stanton et al., 2022).

Despite success in reducing antimicrobial usage (AMU) in some countries (Lam et al., 2017; DANMAP, 2022), AMU is anticipated to rise around the world. Antimicrobial consumption in food-producing animals is projected to reach over 100,000 tonnes per annum by 2030, a 67% increase since 2015, with an estimated 99,502 tonnes used in 2020 (Van Boeckel et al., 2015; Tiseo et al., 2020; Mulchandaniid et al., 2023). In the United Kingdom alone, the use of CIAs on pig farms doubled from 2015 to 2019. The use of aminoglycosides, deemed critically important, rose from 2.607 to 5.957 mg per kilogram of body weight in pigs (Mahase, 2021). In the United States of America, 54% of antimicrobials used for livestock are CIAs. After a reduction in the use of CIAs by 27% from 2009 to 2017, this rose again by 8% from 5.6 million kilograms in 2017 to 6.0 million kilograms of antibiotic active ingredient in 2020 (US Food and Drug Administration Center for Veterinary Medicine, 2020).

The problem with AMR is that it knows no country boundaries, so reducing it globally is essential (Ruckert et al., 2020). At the global and country levels, there are varying efforts and interventions to preserve the repertoire of antimicrobials available for human use and to reduce the development and spread of AMR. The quadripartite, consisting of the World Health Organisation, Food, and Agriculture Organisation of the United Nations (FAO), World Organisation for Animal Health (WOAH), and United Nations Environment Programme (UNEP) calls for a reduction in antimicrobial usage (AMU) and AMR while enhancing antimicrobial stewardship (AMS), within the human medical and animal industry (FAO UNEP WHO and WOAH, 2022). This will mean enhanced research and understanding, One Health collaboration, and implementation of action plans to ensure best practices globally (FAO UNEP WHO and WOAH, 2022).

Working across sectors to reduce AMU and the development and transmission of AMR is essential to reduce the increasing burden and mortality attributed to AMR. Within human medicine, most AMR interventions are implemented in high income countries, with only, an estimated, 1 – 2% focusing on low- and middle-income countries (LMICs) (Cox et al., 2017). A similar trend likely occurs in the animal health sector. In high income countries such as The Netherlands and Denmark, interventions at the national levels have contributed to reductions in AMU in the animal health sector. For example, The Netherlands implemented a strategy aimed primarily at farmers based on the RESET mindset (rules and regulation, education, and information, social pressure, economics, and tools) to reduce AMU and the use of HPCIAs (Lam et al., 2017). This included obligatory aspects such as transitioning from the use of HPCIAs to less critical antimicrobials, having a registered herd veterinarian to discuss herd health with, and voluntary aspects such as lectures and study groups for farmers and animal health professionals (AHPs). This intervention resulted in a reduction in AMU of 47% between 2009 to 2015 (Lam et al., 2017). In Denmark, since 1995 DANMAP, the Danish Integrated Antimicrobial Resistance Monitoring and Research Programme, has monitored both AMU (grouped by antimicrobial class) by farmers, AHPs, and human medical professionals and AMR in both animals and humans. Coupled with other initiatives, DANMAP has led to an overall reduction in prescriptions and use of HPCIAs within all livestock sectors and eradication of HPCIA use within pig production. The monitoring of AMR of indicator bacterial isolates has shown a fluctuating trend for different bacteria. Full sensitivity to antimicrobials increased in *E.coli* isolated from broilers during 2014 – 2019, but the upward trend was not the same for pigs and cattle (DANMAP, 2022).

The burden of AMR is unevenly distributed across the globe. In 2019, high income countries had nearly 50% fewer deaths attributed to AMR (13.0 out of 100,000 deaths) compared to Africa which had the highest rate globally (23.7 out of 100,000 deaths), nearly 1.5 times higher than the global average (16.4 out of 100,000 deaths) (Murray et al., 2022). With a rising middle class and growing population in LMICs, there is increased demand to intensify food production which can lead to higher levels of AMU to sustain the high level of production (Manyi-Loh et al., 2018). The increased intensive farming in LMICs highlights the importance of identifying viable solutions to reduce AMU in livestock production.

There is limited data on existing interventions on AMR, AMS, and AMU in the animal health sector, particularly in LMICs. A few studies attempted to explore these aspects, but the scope was narrow. The aspects investigated in previous review studies included AMS in AHPs (Gozdzielewska et al., 2020), resistance genes within broiler production (Becker et al., 2021), and levels of transmission of AMR to humans after AMU in animals (Tang et al., 2017). An understanding of existing interventions focused on reducing AMU and AMR and increasing AMS within AHPs, farmers, and livestock, globally and in LMICs, is important. To address this gap, we have undertaken a scoping review with the intent of providing a broad overview and categorisation of interventions about AMR in the animal health sector within the last decade. The scoping review provides an overview map of existing evidence on AMR interventions in the animal health sector, and the related impact, current gaps, and limitations. Findings from the review can be used to inform and shape new interventions and to tailor future research on AMR interventions.

## 2 Methods

### 2.1 Study approach

A scoping review was conducted following the PRISMA-ScR checklist (Tricco et al., 2018). The focus of the review was AMR interventions in the animal health sector, specifically interventions aimed at reducing inappropriate AMU, increasing/enhancing uptake of AMS, and/or reducing the risk of development and spread of AMR. The groups of interest to which the AMR interventions were applied included AHPs (veterinarians and para-veterinarians), farmers, and livestock (poultry, cattle, goats, sheep, swine, and aquaculture).

### 2.2 Data sources

The databases PUBMED, Scopus, and Web of Science were searched (Appendix 1). The websites of the World Health Organization (WHO), Food and Agriculture Organization of the United Nations (FAO), and World Organisation for Animal Health (WOAH), were also searched. Backward citation tracking was performed on articles including reviews that were otherwise excluded.

### 2.3 Search strategy, inclusion, and exclusion criteria

A combination of words relating to Africa, America, Animal, Antibiotic, Antimicrobial, Asia, Australia, Bacteria, Environment, Europe, Farmer, Intervention, North, Para-veterinarian, South, Surveillance, Veterinarian, and Veterinary was used for the article search (**Appendix 1**).

No limits were set on study design, the language was set as English and only papers published from the 1^st^ of January 2013 through 31^st^ of December 2022 were included. Reviews were excluded but were accessed for citations. Studies focusing on interventions at various levels were included: global, continent, country, regional (area within a country), or small-scale (multiple or singular herds or farms). Studies were excluded if they solely focused on human, environmental, or human and environmental aspects. Single cross-sectional studies, focusing on opinions or current practices only, and reports with a focus on providing singular time point surveillance data with no intervention action were excluded.

### 2.4 Study screening

The article search was performed in January 2023. Titles and abstracts of identified records were screened against the above inclusion and exclusion criteria. Eligible records were exported to Mendeley and then screened by the author (AJ). Articles that met the inclusion and exclusion criteria were included in the review. References from the full-text searches of articles deemed relevant were also screened against the inclusion and exclusion criteria and included in the review if relevant.

### 2.5 Data extraction

**Figure 1:**
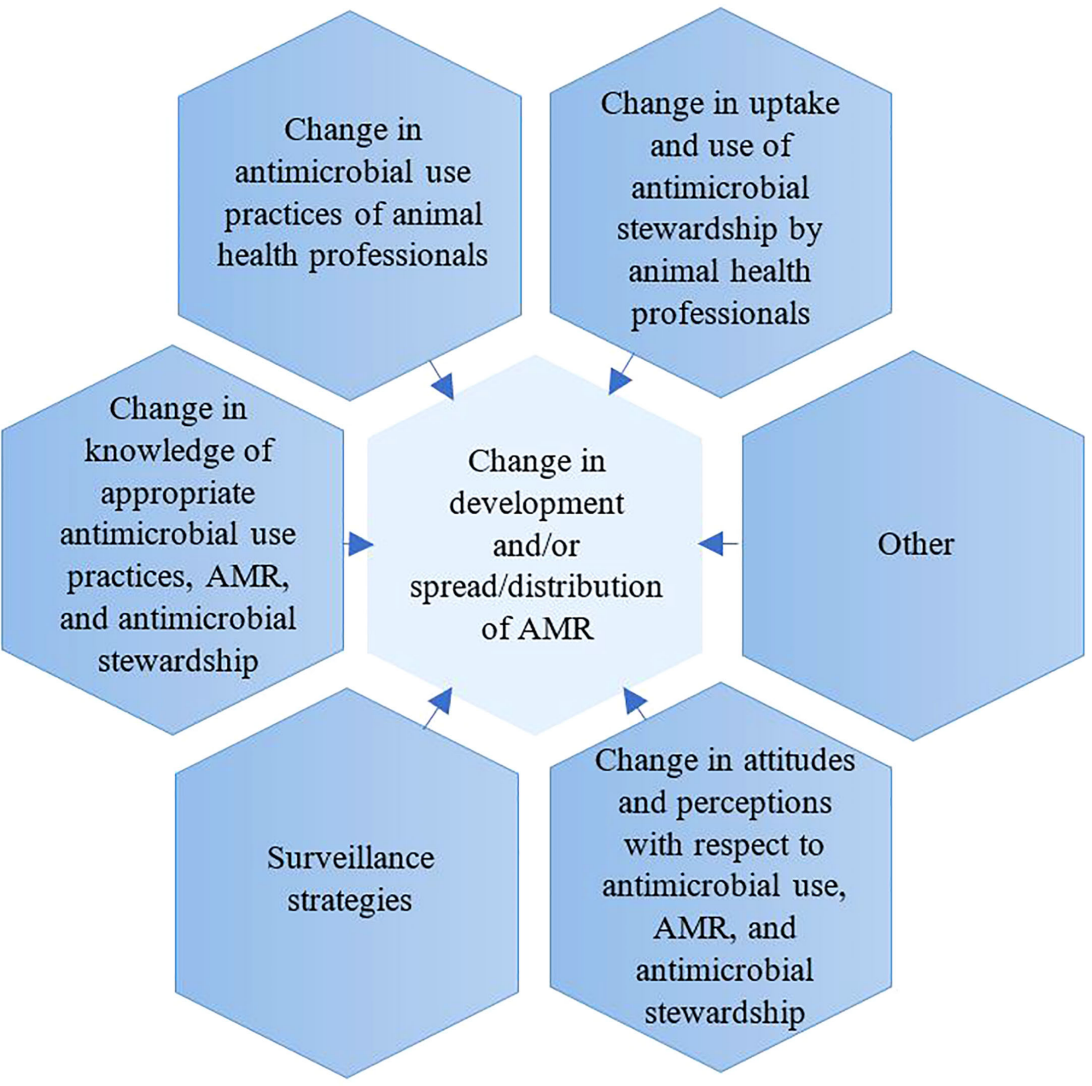
Primary outcome measures.

For each article, the following data were extracted into an excel file: author, year of publication, country where activity was implemented, level of intervention (small scale, regional, national, continental, international), study design, study population (AHPs, farmers, or livestock), results relevant to the primary intervention outcome measures (**Figure 1**, **Appendix 1**), outcomes of the intervention, impact of the interventions, strengths, and limitations. Extraction was performed by one reviewer (AJ) for all eligible articles and a second reviewer (AE) evaluated a subset of 20% of the extracted articles.

### 2.6 Synthesis and reporting results

A key aim of this study was to characterise the reported AMR interventions in the animal health sector, which focused on either livestock or AHPs or farmers. For the purpose of this study, interventions were grouped into seven categories based on primary outcome measures: 1) change in AMU practices of animal health professionals (AHPs) and farmers, 2) change in the uptake of AMS by AHPs and farmers, 3) change in development and/or spread/distribution of AMR, 4) change in knowledge of appropriate AMU practices, AMR and AMS, 5) change in attitudes and perceptions concerning AMU, AMR, and AMS, 6) surveillance strategies (with a focus on animals and either both or one of the following: environment, and/or humans), and 7) Other. For each of the above seven categories, primary and secondary outcome measures were defined (**Figure 1**). Details of defined primary and secondary measures are provided in **Appendix 2**. For purposes of interpretation, the reported interventions were also categorised by level of the geographical area covered as follows: small-scale (e.g., (i.e., singular or multiple herds or farms), regional (region within a country), national (country-level), continental (across an entire continent), and international (across continents) interventions. Due to this review article being a scoping review, and the variety of study designs, a light touch study design assessment was performed.

### 2.7 Study design appraisal

For intervention studies, a light touch quality review of the study design and subjectivity of outcome measurements was performed within the research group (AJ & JO) by asking the questions: (1) what design was used for the intervention? and (2) were the outcome measurements subjective or objective? Interventions were evaluated on a six-point scale based on the above two questions. For question 1, a maximum of four points could be obtained for study design: use of randomisation with control group (4 points), control group with no randomisation (3 points), two time points at which the intervention was measured (pre and post intervention) and with no control group (2 points), description of intervention without use of pre-and post-intervention measurement and no control group (1 point). For question 2, a maximum of two points could be obtained: including objective outcome measurements (2 points) or only subjective outcome measurements (1 point). No points were obtained if outcomes were not included. Outcome measurements were considered objective if were directly measurable (e.g., AMU, AMR genes, bacterial strains) and considered subjective if there was potential bias in reporting by participants (e.g., self-reporting of change). The interventions were split into 3 categories, high, medium, and low quality, based on the combined points (maximum of six points): high = 5 or 6 points, medium = 3 or 4 points, or low = 1 or 2 points. Surveillance reports were not included in this intervention design appraisal.

## 3 Findings

**Figure 2:**
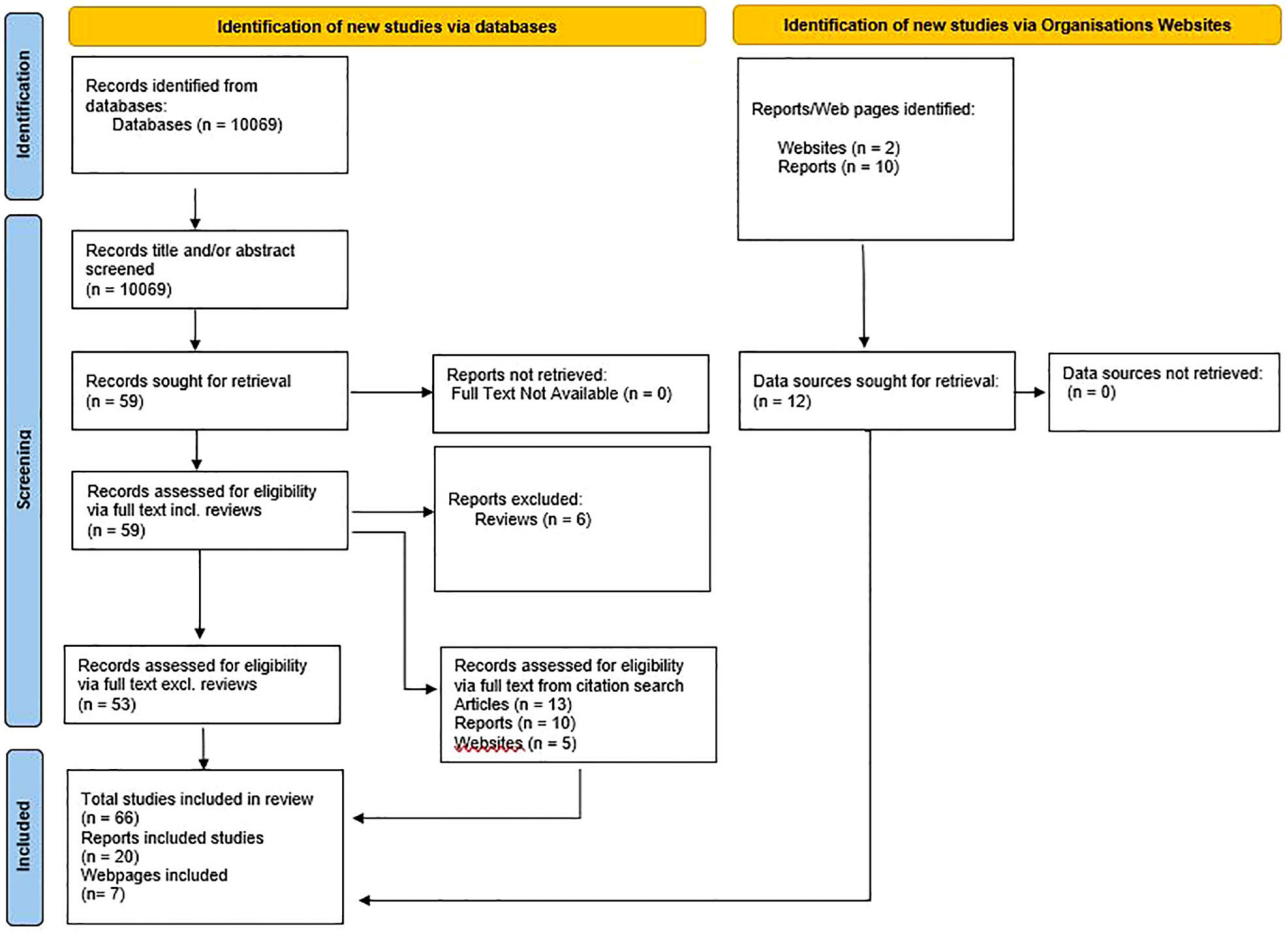
Prisma-ScR Flowchart.

The database search identified 10069 articles, including duplicates, for inclusion. After title and abstract screening, 59 articles were deemed to fall within the inclusion criteria (**Figure 2**). Among the 59 articles duplicates were checked for and none were found. Of the 59 articles, 57 were successfully retrieved and 2 that were not available from online databases were accessed through intra-library loans. Six of the 59 articles were reviews and were therefore excluded resulting in 53 eligible articles. Through citation search, 28 additional sources were found (13 articles, 10 reports, and 5 webpages). International Organisation websites were searched for interventions that fit within the scope of the primary outcome measures and 10 reports and 2 webpages were included. In total 93 sources were included – 66 articles, 20 reports, and 7 webpages (**Figure 2**).

**Figure 3:**
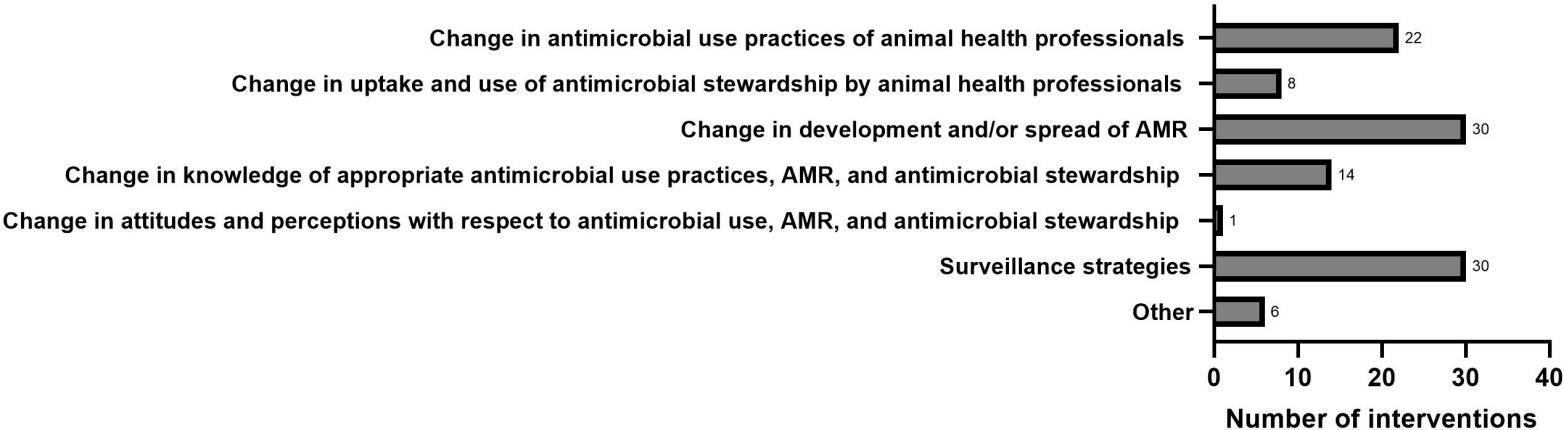
Interventions grouped by primary outcome measurements. Interventions are counted in more than one group if they incorporated more than one primary outcome measurement.

The ninety-three studies and reports resulted in 90 interventions (66 articles, 20 reports, and 7 webpages. The distribution of primary outcome measures for interventions assessed in this study was broad (**Figure 3**), with some overlap between measures. The reported interventions focused mostly on surveillance strategies (30/90), change in development and/or spread of AMR (30/90), change in AMU practices (22/90), change in knowledge (14/90), and change in the uptake of AMS (8/90). Studies reporting change in attitude and perceptions were nearly non-existent (1/90). Six (6/90) sources were categorised as ‘Other’.

The interventions were implemented at various levels including small-scale (singular or multiple herds or farms), regional (region within a country), national (country-level), continental (across an entire continent), and international (across continents). Most interventions were implemented on a small scale (51/90) or national/country level (24/90), with fewer on an international (across continents) (7/90), continental (4/90), or regional level (area within a country) (4/90).

National interventions mostly took place in countries in Europe (14/24), North America (5/24), and Asia (5/24), whereas all continental interventions took place in Europe (4/4). Small-scale interventions were mostly implemented in countries in Europe (23/51), North America (14/51) and Asia (8/51). Of the country level studies, 16/83 were performed in LMICs (international studies are excluded from the denominator).

Intervention design appraisal was performed on 62/90 of the included studies. Of these, 25/62 were categorised as high quality, 32/62 were of medium, and 5/62 of low quality (**Tables 1**–**7**). The distribution of studies based on design quality in LMICs was high (2/13), medium (9/13), and low (2/13).

**Table 1:** Change in antimicrobial use practices of animal health professionals.

Size	Source	Tool/Intervention	Target population	Secondary outcome	Outcome	Impact	Strengths	Limitations	Location	Quality Grade
Continental	The European Parliament and The Council of the European Union, 2022	Regulation (EU) 2019/6 on veterinary medicinal products and repealing Directive 2001/82/EC.	Farmers, AHP	Area	Ban on preventative AMU to groups or via food, reinforce AMU for growth promotion is banned.	Highlights the need for methods to reduce AMU.	Not voluntary - easier to incentive/create repercussions for non-compliance.	Only used within the EU.	Europe	N/A
National	Agunos et al., 2017, Agunos et al., 2018	Canadian Integrated Program for Antimicrobial Resistance Surveillance.	AHP, Broilers, Turkey	Area	Between 2003 - 2015, ceftiofur resistant *Salmonella* decreased by 7 % at farm level. Ceftiofur resistant *E. coli* decreased by 16%, 11%, 8 %, in farm, abattoir and retail samples respectively.	Reduction in ceftiofur resistant *Salmonella* and *E. coli*. Increase in *E. coli* resistance to gentamycin and S/TMP.	Long time frame, all testing performed at national reference lab.	No detailed risk factor analysis	Canadian	Medium
National	Bradley et al., 2017	Same intervention as Breen et al, 2017. National mastitis control scheme: AHDB Dairy Mastitis Control Plan (DMCP) (includes surveillance and actions).	AHP, Farmers & Cattle	Area, DDx	Multiple outcomes around AMU and AMS incl. 400 AHP and Farmers trained. 40% reduction in intramammary lactating cow use. 20 % reduction in clinic mastitis rates.	Increased training and knowledge for farmers and AHPs, reduced AMU for dairy cattle.	No evidence presented.	Letter in journal, no evidence or evaluation.	United Kingdom	Medium
National	Breen et al., 2017	Same intervention as Bradley et al, 2017. National mastitis control scheme: AHDB Dairy Mastitis Control Plan (DMCP) (includes surveillance and actions).	AHP, Farmers & Cattle	Area, DDx	Reduction from Total DDD of 14.59 to 6.99 in 600 dairy cattle herd.	Reduction of AMU in Dairy herd.	Easy to follow control plan, using parameters that are already evaluated as reference.	Single herd example.	United Kingdom	Medium
National	Dupont et al., 2017	Yellow card scheme. Same intervention as Jensen et al., 2014.	AHP, farmers, swine	Area	38.4 - 56.2 % reduction mg active ingredients/pig/day (37.2 - 53.6 % reduction in ADDs/100pigs/day). Biggest perceived factors: Vaccines increased; herd medication decreased	AMU reduction in both high and low usage herds.	National programme (large sample size, even with exclusions).	Factors only measured in herds with >10 % antimicrobial reduction.	Denmark	Medium
National	Jensen et al., 2014	Yellow card scheme. Same intervention as Dupont et al., 2017.	AHP, farmers, swine	HPCIA	27 % (weaner) and 53 % (finisher) reduction ADDD25 per pig produced of macrolides and pleuromutilins 2009 - 2011	Reduction of AMU HPCIA.	National programme (large sample size, even with exclusions).	Short time frame compared to Dupont et al., 2017	Denmark	Medium
National	Moura et al., 2022	Vet-AMNet.	Livestock AHPs Farmers	Area, HPCIA	Reduction of 70.8% kg in AMU.	Reduced AMU nationally in Netherlands for dairy cattle.	National project - both voluntary and legislation.	Article looking to validate system, not outcomes.	Netherlands	Medium
National	Phu et al., 2021	ViParc (Education of small-scale broiler Farmers, antimicrobial replacement products, designated project veterinarian for herds).	Farmers, Broilers	Herd	66% decrease in AMU (p=0.002) from a baseline of 343.4 Animal Daily Doses per 1,000 chicken-days.	Reduction in AMU while decreasing mortality and increasing body weight of broilers.	Simple illustration of educational intervention.	Not evaluated on intensive farming. Only applicable for small scale farms.	Vietnam	Medium
Regional	Millar et al., 2022	Regulation restricting use of HPCIAs in animals.	Farmer, AHP, cattle	HPCIA	HPCIA reduction from 14,258 - 21,528 Canadian Defined Course Doses for cattle (DCDbovCA) /month to a range of 1,494 - 4,707 DCDbovCA/month (Sales data).	Significant reduction of HPCIA on dairy herds.	Large sample size.	Sales data does not show actual usage. No reflection on potential increase in other AMU.	Quebec, Canada	Medium
Small Scale	Becker et al., 2020	Outdoor Veal: 1) Transported directly to farm with no intermingling, 2) Vaccination against pneumonia and 3-week quarantine, 3) Raised in hutches with max 10 cattle.	AHP, cattle	Herd	Treatment intensity defined daily dose method (TIDDD) in days per animal year was 5.3-fold lower (5.9±6.5 vs. 31.5±27.4 days per animal year; p<0.001) than control group.	AMU reduced significantly in outdoor veal calf herds.	Reduced AMU, mortality, and no compromise to animal health.	Requires flat land and access to isolation transport and hutches. No mention of costs.	Switzerland	High
Small Scale	Collineau et al., 2017	Herd specific intervention plans.	Farmer, Swine	Herd	Median 47% reduction in AMU (treatment incidents), without increased mortality. No correlation to type or number of interventions.	Reduction of AMU across countries and intervention types.	Big variation in net profit post intervention.	Personalised intervention: time and economy considerations need for broader scale.	Belgium, Germany, France, Sweden.	Medium
Small Scale	Dorado-García, Dohmen, et al., 2015	1) AMU reduction 2) Improving personnel and farm hygiene 3) Change animal contact structures.	Farmer, Swine	Herd	See *Table 3: Change in development and/or spread/distribution of AMR.*				Netherlands	Medium
Small scale	Gerber et al., 2021	17 interventions from three groups 1) Udder 2) Uterine health 3) Calf health. Farmers had to pick at least one.	Farmer, Cattle	Herd, DDx, HPCIA	AMU reduced by an udder or uterine health strategy (p < 0.04), including HPCIA for udder strategies (p = 0.05). Calf health interventions no reduction.	Uterine and udder strategies saw a reduction in AMU and HPCIA while calf strategies did not.	Gave Farmers choice in intervention.	Intervention vs. control group vastly different in breed, herd size and milk yield (selection bias across both).	Switzerland	High
Small scale	Gomez et al., 2021	Management modification, health training and algorithm.	Farmer, Cattle	DDx	AMU reduced from 85% to 18% of diarrhoea calves being treated with AM, with mortality and diarrhoea incidence staying the same.	AMU reduced significantly in outdoor veal calf herds.	Included education – this exists beyond intervention frame.	Control was prior to intervention, not concurrent.	Ontario, Canada	Medium
Small scale	Gomez et al., 2017	Two algorithms looking at calf diarrhoea and AMU incidence rate in herd.	Farmer, Cattle	DDx	Cumulative Incidence Risk (CIR) of antimicrobial treatment rates 80% lower after implementation.	Use of the algorithm reduced incidence of AMU.	Good sample size, replicated. Algorithm can be used elsewhere.	The two algorithms did not run concurrently.	Ontario, Canada	Medium
Small scale	Kuipers et al., 2016	Actively guided use of antibiotics, biannual meetings with project members and a veterinarian, AMU feedback reports.	Farmers, cattle	Herd, DDx, Area	ADDD reduced earlier in study for guided group than control (n=2) groups & less overall use. Mean guided = 5.45. Control groups = 6.34 & 5.63. Variation between herds decreased.	Reduction in AMU and differences both pre-post study but also in control groups.	Case control study, taking trends in time into account.	Role of herd health and veterinarian not analysed as a factor.	Netherlands	High
Small Scale	Morgans et al., 2021	Facilitated farmer action groups.	Farmers, cattle	HPCIA	See *Table 4: Change in knowledge of appropriate AMU practices, AMR, and AMS*				United Kingdom	Medium
Small scale	Pempek et al., 2022	AMS training for farmers in two parts: didactic presentations, calf-side training, and veterinarian feedback.	Farmers, Cattle	Herd	Increased knowledge in post intervention test compared to pre-test and control group (CG) (p= 0.05). Correct identification of cases 50% (73/146) of the cases vs. 14.3% (9/63) in GC (p= 0.002). (Increased later in intervention compared to earlier also (p< 0.001). 50 % decrease in AMU compared to CG.	Increased understanding of AMS, increased correct identification of cases’ need for AMU, and decrease in AMU	Holistic approach. "Test" to evaluate increased understanding, rather than farmer perception.	Cannot randomly allocate farms. Bias due to farms with interest in AMS/AMR. Small sample size.	Ohio, USA	High
Small Scale	Postma et al., 2017	Same intervention as Rojo-Gimeno et al., 2016. Herd specific intervention plans (including herd management, biosecurity, vaccination strategy, anti-helminthic therapy, and AMU).	Swine, Farmers	Herd, HPCIA	Decrease of 52% in AMU (from birth till slaughter) and 32% for breeding animals, based on treatment incidences. Ceftiofur long-acting AMU in sucklers reduced 83%.	Decrease in AMU and HPCIA.	Looked at AMU for different age groups. Personalised interventions that work for individual herds.	Veterinarians reluctant to provide information on curative AMU.	Belgium	Medium
Small scale	Raasch et al., 2020	Herd specific intervention plans (includes biosecurity, vaccination, changes of feeding schemes or drinking water quality, health and welfare care, stable climate and zootechnical measures).	Farmers, Swine	Herd, HPCIA	93% median compliance of participants. Median 35% reduction in AMU treatment in % of expected lifespan. Reduction from 35 % to 16 % (p< 0.001). HPCIA reduced 69% polymyxin p < 0.001. Tetracycline 49% (p = 0.01).	Decrease in AMU and HPCIA.	Looked at AMU for different age groups and DDx. Personalised interventions that work for individual herds.	Control herd also changes over a year period, therefore own control. External factors impact over a year. Farmer with AMR interest participate.	Belgium Germany France Sweden	Medium
Small Scale	Rojo-Gimeno et al., 2016	Same intervention as Postma et al., 2017. Herd specific intervention plans (including herd management, biosecurity, vaccination strategy, anti-helminthic therapy, and AMU).	Farmers, swine	Herd	Median reduction of 7.68 euro/sow/year spent on AMU prophylaxis.	Increased net profit while reducing AMU.	Profit focused, economic trade off important for farmers.	Little data on antimicrobial groups and usage of these. No follow up post intervention, veterinarians reluctant to provide curative AMU data.	Belgium	Medium
Small Scale	Schreuder et al., 2022	Health plan and improved biosecurity personalised, multiple intervention cycle.	Farmers, broilers	Herd	A number of farms did not use any antimicrobial after intervention cycle 1 (n = 4) and 2 (n=5). Mean days of treatment pre-post intervention cycles did not change in any country.	Reduction in AMU on some farms in Cyprus but mean days of treatment stayed the same in all countries.	Some comparison and reflection between countries, impact of personalisation varies.	Only descriptive statistics for AMU.	Netherlands, Greece, Cyprus	Medium
Small Scale	Speksnijder et al., 2017	Animal Health Planning Program.	Farmers, Cattle	Herd	DDDA of antimicrobial - 19% vs. 14% in control group after 1 year.	No significant difference (intervention vs. control) in AMU reduction (P = 0.498).	Farm selection was from group with higher AMU load “signalling zone”. Good reflection on needing real world reduction, potentially reducing participant bias.	AMU was measured via prescription not usage, does not account for wastage/stockpiling. Participating bias if interested in AMU/AMR.	Nederlands	High
Small scale	Toya et al., 2022	Intervention in 3 parts: 1) awareness of AMR, 2) consent for diagnosis and treatment, and 3) Reduce AMU.	Farmers, cattle	Herd	DDD/slaughter pig 43% post intervention of what it was pre intervention (910.2 vs. 397). Non-intervention farms 146.2% (531 vs. 777).	Decrease for all indicators on intervention farms, while there was an increase on control farms.	Study had both a control group and pre-post intervention assessments.	Small sample size, bias due to voluntary participation.	Japan	High
Small Scale	Turner et al., 2018	Farmer meetings, herd health planning meeting, change of treatment protocols, no HPCIAs without prescriptions.	Farmers, swine	HPCIA	HPCIA on farms reduced from 7.2 ADD (41%) to none.	Complete cessation of HPCIA use on farms.	Multifaceted approach.	All farms under care of one veterinary practice.	United Kingdom	High

AMU, Antimicrobial use.

AMR, Antimicrobial resistance.

AMS, Antimicrobial stewardship.

AHP, Animal Health professional.

ADD, Animal daily dose.

ADDD, Animal defined daily doses.

DDD, Defined daily dose.

DDDA, Yearly moving average total antimicrobial use.

E. coli, Escherichia coli.

Herd, Reduction of volume/weight of AMU on herd level.

DDx, Reduction of volume of AMU for a specific diagnosis.

Area, Reduction of volume of AMU for livestock animals on regional, national, or continental level.

HPCIA, Reduction in volume of use of Critically Important Antimicrobials for Human Medicine.

Sulfa/TMP, Sulfadiazine/Trimethoprim.

**Table 2:** Change in uptake and use of antimicrobial stewardship by animal health professionals.

Size	Source	Tool/Intervention	Target population	Secondary outcome	Outcome	Impact	Strengths	Limitations	Location	Quality Grade
Continental	The European Parliament and The Council of the European Union, 2022	Regulation (EU) 2019/6 on veterinary medicinal products and repealing Directive 2001/82/EC.	Farmers, AHP	Px	See **Table 1**: Change in antimicrobial use practices of animal health professionals.				Europe	N/A
National	Dupont et al., 2017	Yellow card scheme.	AHP, farmers, swine	Px, Other	See **Table 1**: Change in antimicrobial use practices of animal health professionals.				Denmark	Medium
Regional	Abdelfattah et al., 2022	Senate Bill 27 - Prescription required for medically important antimicrobials.	Farmers, AHP	Diagnostic, Other	Self-reported: 29.4% changed disease management, 26.8 % report using antimicrobial preventative alternatives.	Reported increases in preventative alternatives and changed disease prevention/management.	Law bill, not voluntary.	Low response rate & most likely only those interested in AMS responded. Did not report factors within categories or actual AMU changes.	California, USA	Medium
Regional	Rees et al., 2021	The Arwain Vet Cymru Project - Veterinary Prescribing Champions (VPC) (incl. webinars, workshops, discussion).	AHP	Px, guidelines	See **Table 4**: Change in knowledge of appropriate AMU practices, AMR, and AMS.				Wales, United Kingdom	Low
Small Scale	Morgans et al., 2021	Facilitated farmer action groups.	Farmers, cattle	Other	See **Table 4**: Change in knowledge of appropriate AMU practices, AMR, and AMS.				United Kingdom	Medium
Small Scale	Musoke et al., 2020	One Health training - knowledge on AMR, sanitation (case studies, group discussions).	AHP, MHP	Px, guidelines, diagnostics, Other	Of health professionals (%) reported improved: handwashing (57.3 %), guideline use (52.9 %), treatment based on diagnostics (44.1%) + reduction in unnecessary AMU (51.3 %).	Improved practices and knowledge of AMS.	One Health approach, part of already existing structure.	Low participation of AHP compared to MHP. Self-reporting of improvement.	Uganda	Low
Small scale	Pempek et al., 2022	AMS training for farmers in two parts: didactic presentations, calf-side training, and veterinarian feedback.	Farmers, Cattle	Other	See **Table 1**: Change in antimicrobial use practices of animal health professionals.				Ohio, USA	High
Small Scale	Roulette et al., 2017	Knowledge and innovations for: 1) prudent AMU (tape measures & dosage charts (calculate weight for more accurate dosage), 2) pasteurization milk (thermometers) to reduce resistant E. coli.	Farmers	Other	See **Table 4**: Change in knowledge of appropriate AMU practices, AMR, and AMS.				Tanzania	Medium

AMU, Antimicrobial use;

AMR, Antimicrobial resistance;

AMS, Antimicrobial stewardship;

AHP, Animal Health professional;

MHP, Medical Health Professional;

Px, Change in prescribing habits (define the prescribing habits for which change will be measured).

Guidelines, Increased adherence to guidelines.

Diagnostics, Increase in frequency of use of diagnostics e.g., sensitivity testing.

**Table 3:** Change in development and/or spread/distribution of AMR.

Size	Source	Tool/Intervention	Target population	Secondary outcome	Outcome	Impact	Strengths	Limitations	Location	Quality Grade
National	Agunos et al., 2017, Agunos et al., 2018	Canadian Integrated Program for Antimicrobial Resistance Surveillance.	AHP, Broilers, Turkey	B strain, R strain, Area	See *Table 1: Change in antimicrobial use practices of animal health professionals.*				Canada	Medium
National	Hiki et al., 2015	Voluntary reduction of ceftiofur in hatcheries.	Broilers	R strain	Reduction of cephalosporin resistant *E. coli* from 16.8% (27/161) to 4.6% (6/131) (p=0.001).	Reduction of cephalosporin resistant *E. coli* after ceftiofur reduction.	Assessed multiple resistance genes.	Longitudinal study. No control group to assess for confounding. No assessments of mortality or animal health.	Japan	Medium
Small scale	Bahrndorff et al., 2013	Fly screen placement on broiler houses, which remove 95% of fly population.	Broilers	B strain	Reduction in prevalence of *Campylobacter spp* in flocks from 41.4 % to 10.3 % (p < 0.001). (Control house reduced from 41.8 % to 36 % (p = 0.454).	Reduction in prevalence of *Campylobacter spp* among flocks.	4-year data set, use of a control.	No data collected on poultry disease levels or end meat product.	Denmark	Medium
Small Scale	Brusa et al., 2019	Reducing Shiga toxin gene in hide samples, washing abattoir pens using 1) electrolytically generated hypochlorous acid, and 2) chlorinated water, electrolytically generated hypochlorous acid, and isochlor.	Cattle	R strain	1) Pre - post intervention 96.6% vs. 16.6% positive samples (p < 0.001) 2) 9.4 times less risk of positive sample post intervention (p = 0.003).	Reduction of Shiga toxin in samples post intervention.	Used steers from high intensity breed farms to increase probability of high bacterial load.	High chlorine levels might not be allowed in some countries. Small sample size.	Argentina	Medium
Small Scale	Ceccarelli et al., 2017	Aviguard (probiotic) given to two chick groups: infectious and susceptible.	Broilers	R strain	Excretion: 1.17 CFU/g faeces (infectious and susceptible chicks) vs. control 5.68 CFU/g (p < 0.001).	Statistically significant reduction in transmission + excretion of ESBL- *E. coli.*	Tested every day. Multiple scenarios used.	Only tested for 13 days after ESBL-*E.coli* given.	Netherlands	High
Small Scale	Chinwe et al., 2014	No antibiotic feed additives given and assessed for *E. coli.*	Broilers	R strain, B strain	*E. coli* in cloacal swabs was 11% lower (17%) in flocks with no antimicrobial feed additives. Higher number of susceptible *E. coli* isolates across all antimicrobials assessed.	Less prevalence of *E. coli* in cloacal samples and resistant *E. coli* isolates.	Trialled in LMIC farm environment.	The reported data on carriage is not clear/confusing.	Nigeria	High
Small Scale	Cicconi-Hogan et al., 2014	Organic certification.	Cattle	R strain	Methicillin resistance coagulase negative staphylococci in 2 % of organic and 5 % of conventional bulk tank milk, MRSA in 0.3 % organic.	Reduced prevalence of methicillin resistance coagulase negative staphylococci in organic bulk tank milk.	Provides data on AMR specific (MRSA) information.	Few farms from each area, very general overview. Only Farmers with interest (already low levels).	USA	High
Small Scale	Dame-Korevaar, Fischer, et al., 2020	Competitive exclusion: 1) fermented intestinal bacteria (CEP), 2) selection of pre- and probiotics (SYN).	Broiler, chicks	R strain	Challenge on day 0 CEP + SYN no effect. Challenge day 5 CEP + SYN prevented CTX-M-1-*E.coli*. excretion (up to -1.60 log10cfu/g), and caecal content (up to -2.80 log10cfu/g).	Competitive exclusion reduced prevalence of CTX-M-1-*E.coli.*	Trialled different challenge points - day 0 and 5.	No data collected on poultry disease levels or end meat product.	Netherlands	High
Small Scale	Dame-Korevaar, Kers, et al., 2020	Competitive exclusion in semi-field conditions.	Broiler	R strain	0/200 broilers CTX-M-1-*E. coli* positive on day 21 vs. Control 187/200 positive.	Competitive exclusion reduced prevalence of CTX-M-1-*E.coli*.	Detailed microbiota composition.	Performed with stringent biosecurity, outcome might vary by field environment.	Netherlands	High
Small Scale	Dorado-García, Dohmen, et al., 2015	Intervention with multiple steps: 1) reduce AMU, 2) improving personnel and farm hygiene, and 3) change animal contact structures.	Farmer, Swine	R strain	44% decrease in AMU (DDDA/Y) and decrease of MRSA positive farms from 31 to 29.	Reduced AMU and MRSA positive isolates, with a correlation to avoiding teeth clipping and keeping sows in stable groups.	Assessed multiple factors and retested over 4 intervals of intervention.	Pooled samples in testing, short time frame for MRSA.	Netherlands	Medium
Small Scale	Dorado-García, Graveland, et al., 2015	Two intervention groups: 1) Reducing AMU with protocol (RAB) and 2) RAB + Cleaning and disinfection (CD). Testing for MRSA.	Cattle	R strain, environment	2 - 3 times higher level of MRSA in veal cattle in Control & RAB-CD than RAB at 12 weeks in both cycles of intervention (p value = 0.5 and < 0.01, 1^st^ and 2^nd^ cycle respectively). Human nasal samples not statistically significant, and environmental samples were negatively impacted by CD.	Statistically significant lower levels of MRSA in RAB cattle intervention population but not human workers.	Multiple areas of swabbing within populations to see if reduction in cattle reflects in human workers.	Different sample techniques in first and second cycle. Only 12 weeks of intervention.	Netherlands	High
Small Scale	Hao et al., 2013	Slightly acidic water for *E. coli* and *Salmonella* reduction (pH 5.0 - 6.5) with chlorine concentration (300mg/L).	Broilers	B strain, Environment	Number *E. coli* and *Salmonella* positive swabs reduced by 16%.	Reduction in presence of *E.coli* in broiler house.	Swabbing large range of area.	Intervention not feasible on cage floor.	China	Medium
Small Scale	Haskell et al., 2018	RWA for MRSA.	Poultry, Cattle, swine	Food	15.7% of conventional raw meat samples contain MRSA, 0% of RWA turkey or chicken contained MRSA. However, increased level of MSSA.	No MRSA isolates from RWA turkey and chicken retail meat.	Intervention had high success rate for MRSA even with some MSSA prevalence.	Only tested RWA turkey and chicken, small sample size. Does not reflect worker contamination in relation to MRSA prevalence in herd. RWA comes with ethical issues.	Utah, USA	High
Small Scale	Kannan et al., 2019	Dietary brown seaweed +/- spray with chlorinated water.	Goat	B strain	Spray wash reduced aerobic plate count of *E.coli* on skin (3.65 vs. 4.30 log10CFU cm^2^). Rumen *E.coli* count reduced with seaweed diet (p < 0.05).	Both seaweed and spray resulted in reduction of *E.coli.*	Range of samples used. Seaweed good food source.	Small sample size, no information to determine if rumen *E. coli* translates to less AMR or AMU.	Georgia, USA	High
Small Scale	Kassem et al., 2017	Organic certification.	Broilers	R strain	Lower presence of ciprofloxacin, erythromycin, and tylosin resistance (p < 0.05) in faecal *Campylobacter* samples.	Reduced presence in *Campylobacter* resistance genes on some farms.	Showed differences between management, and biosecurity.	Only three organic farms investigated and one farm varied widely from other two farms.	Ohio, USA	High
Small Scale	Kempf et al., 2017	Organic certification.	Swine	B strain, R strain	No significant difference in *Campylobacter* in conventional vs. organic in France and Sweden. France: 43/58 (74%); 43/56 (77%) and Sweden: 24/36 (67%); 20/36 (56%). Erythromycin resistance in conventional vs. organic in France: 62 (50%) and 25 (18%).	No significant differences between *Campylobacter* spp but there were differences in AMR gene prevalence.	Illustrates how interventions might work in some countries but not in others.	Organic definition differs depending on country/continent.	France, Sweden	High
Small Scale	Lee et al., 2017	Sanitation and education intervention about cleaning milking equipment and udders.	Farmers, cattle	B strain	40% log reduction of *Staphylococcus aureus* in fresh milk sample.	Reduction of bacteria in fresh milk.	Both qualitative data from farmers and quantitative data from milk.	No statistics on whether statistically significant.	Malaysia	Medium
Small Scale	Loayza-Villa et al., 2021	RWA for two generations.	Swine	R strain	No statistically significant reduction in antimicrobial-resistant coliforms in faecal samples compared to control group.	Two generations of RWA were not enough to see a reduction in antimicrobial resistant coliforms.	Followed more than one generation. Control group. Randomised. Looked at range of resistance genes.	Pooling of faecal samples. Could only follow two generations as pigs sent for slaughter.	Ecuador	High
Small Scale	Luyckx et al., 2015	Cleaning Protocols for *E.coli* (commercial solution containing sodium hydroxide).	Broilers	B strain	Number of *E. coli* positive swabs reduced by 86% (1 - 3% difference depending on soaking & water temperature).	Reduction in presence of *E.coli* in broiler house.	Multiple cleaning protocols and factors assessed.	No differentiating between ESBL and others.	Belgium	Medium
Small Scale	Mourand et al., 2017	*E. coli* pro-biotic strain ED1a	Swine	R strain	Four trials - most comparisons between control and intervention groups showed no statistical significance.	Limited effect on shedding of ESBL-*E.coli*.	Trialled different doses of ED1a, across multiple data points for each group.	Artificial contamination with pathogenic *E.coli* strain. Might not reflect real world situation.	France	High
Small Scale	Methner et al., 2019	Competitive exposure (CE) culture for ESBL and AmpC *E.coli* (EEC).	Broilers	R strain	Difference in EEC between CE culture and untreated controls: 4.0 vs. 5.0 log10 units on day 37 of age.	Reduced load of EEC in birds treated with EC.	Non-invasive, not ongoing. One off colonisation with bacteria.	Birds needs broad range of probiotic strains for protection.	Germany	Medium
Small scale	Nuotio et al., 2013	Competitive exposure (CE) (BROILACT). for ESBL and pAmpC *E.coli*	Broilers	R strain	Reduced prevalence of resistant isolates in ceca samples.	Reduced prevalence of resistance isolates in ceca of chicks.	Checked effect of already implemented existing intervention.	Study only continued 5 days after challenge with *E.coli.*	Finland	Medium
Small Scale	Pedroso et al., 2013	RWA, Pro- and Pre-biotics.	Broilers	R strain	No statistically significant reduction in tetracycline-resistant *E. coli* or class 1 integron resistance element.	No significant difference in resistance isolates between the groups.	Extended length of study. Many aspects were compared.	Two farms only considered; third farm testing not considered.	USA	High
Small scale	Rubio-García et al., 2015	Allostatic modulator in tap drinking water (48h before shipment) with 10h or 16h feed withdrawal for coliforms.	Broilers	B strain, Food	10h feed withdrawal produced 0.29 log10 CFU/ml carcass rinse coliforms. 16h feed withdrawal produced ~0.92 log10 CFU/ml coliforms at carcass rinse	Reduction in coliforms (p = 0.014) and total aerobic mesophilic bacteria (p = 0.0001).	Cost effective intervention. Swabs taken at production give indication of residue in meat.	Testing in experimental setting and not a production setting. Swabs only taken at production not in live birds.	Mexico	Medium
Small scale	Sapkota et al., 2014	Organic certification.	Broilers	R strain	Resistant *Salmonella Kentucky* isolates less prevalent in litter, water, and feed on organic farms: amoxicillin–clavulanate (p= 0.049), ampicillin (p= 0.042), cefoxitin (p= 0.042), ceftiofur (p= 0.043) and ceftriaxone (p= 0.042)	Antibiotic resistant *Salmonella Kentucky* less prevalent on organic farms.	Compared detection of *Salmonella Kentucky* isolates across the farms.	All farms under one feed mill and small area. All control group farms, no pre-post interventions.	USA	High
Small scale	C. Shen et al., 2020	Cessation of colistin as a feed additive to reduce mcr-1 resistance in *E.coli*	Swine	R strain, environment, food	Reduction of mcr-1 on farms (81% to 23% p < 0.0001), in pork (52% to 29%, p < 0.0001) as well as soil and water around slaughterhouses (49% to 27%, p < 0.0001).	Significant reductions in mcr-1-positive *E. coli* after cessation of colistin as a feed additive for swine.	Broad sampling pool both in terms of provinces and sample populations.	Sampled same 3-month period each time so may miss seasonal differences.	China	Medium
Small Scale	Thapaliya et al., 2017	RWA testing for MRSA.	Poultry, Cattle, Swine, Aqua culture	Food	0.4% (n = 2/530) of RWA meats, and 1.4% (n = 39/2760) of conventional meat were MRSA positive.	No statistically significant lower levels of MRSA in RWA meat.	Larger sample size than other RWA meat studies.	Cannot differentiate pre/post slaughter contamination. Limited RWA meat.	USA	High
Small scale	Verrette et al., 2019	Cessation of ceftiofur use from hatcheries to reduce resistance	Broilers	R strain	ESBL/AmpC blaCMY-2 and blaCTX-M genes reduced by 7% and 6%, respectively, in meconium after cessation of ceftiofur, 0% and 20% respectively in faeces of broilers, 0% and 6% respectively in faeces of breeders. However, increased to or above levels prior with introduction of lincomycin-spectinomycin.	Decrease in resistance genes after stopping ceftiofur in ovo but increase after replacement with lincomycin-spectinomycin.	Several testing methods performed.	Whole flocks pooled for sampling, trends.	Canada	Medium
Small scale	Vikram et al., 2017	Non antimicrobial treated cattle (RWA) for a range of AMs	Cattle	R strain	Erythromycin-resistant Enterococcus sp. concentrations in faeces. RWA 61% less than control (p < 0.01).	Reduced erythromycin resistance but not MLS or tetracycline.	Considerable number of resistance genes assessed not just bacterial strains.	Faeces not collected and tested at processing plant to assess risk of exposure/contamination.	USA	High
Small scale	Wanninger et al., 2016	Intervention: RWA testing tetracycline resistance of *Clamydia Suis* (*C. suis*) with 2 controls. Control 1) herd level prophylactic oral AMU (trimethoprim, sulfadimidine, and sulfathiazole (TSS) Control 2) herd treatment with chlortetracycline +/- tylosin and sulfadimidine (CTS).	Swine	R strain	At the start and end of intervention 0% of *C. Suis* isolates resistant in RWA. Control 1) 67% (start) and 0% (end) Control 2) 38% (start) and 83% (end).	Absence of tetracycline treatment led to the absence of resistant insolates.	Provides details on who performed treatments: vet vs. para-veterinarian vs. other.	No evaluation of statistical significance. Small population. Control group 2 was unable to treat *C. suis* on herd level.	Switzerland	High

AMU, Antimicrobial use;

AMR, Antimicrobial resistance;

AMS, Antimicrobial stewardship;

CE, Competitive Exposure;

CFU, Colony forming unit;

ESBL, Extended spectrum beta lactamase;

E. coli, Escherichia coli;

MRSA, Methicillin resistant staphylococcus aureus;

RWA, Raised without antimicrobials;

B strain, Reduced frequency of bacterial strain;

R strain, Reduced frequency of resistance genes within bacterial strain;

Area, Reduced frequency of resistance genes detected/isolated in livestock spp at regional, national, or continental level.

Environment, Reduced frequency of resistance genes detected/isolated within herd/environment around herd.

Food, Reduced frequency of resistance genes detected/isolated in food products (meat, milk, egg, etc).

DDDA/Y, defined daily dosages animal per year.

**Table 4:** Change in knowledge of appropriate AMU practices, AMR, and AMS.

Size	Source	Tool/Intervention	Target population	Secondary outcome	Outcome	Impact	Strengths	Limitations	Location	Quality Grade
International	Food and Agriculture Organization of the United Nations, n.d.-	FAO-PMP-AMR - Help countries to create national action plans (NAPs).		AMU	See *Table 6: Surveillance strategies.*					N/A
International	World Organisation for Animal Health, 2021	OIE Calculation Tool, helps countries calculate AMU.			25% of countries reporting AMU to WOAH use tool to collect AMU product information and calculate active ingredients.	Defined targets for national AMR surveillance in food and agriculture sectors.	Presence of tool.	Limited information about tool.		N/A
National	Bradley et al., 2017	National mastitis control scheme: AHDB Dairy Mastitis Control Plan (DMCP) (includes surveillance and actions).	AHP, Farmers & Cattle	AMR, AMS	See *Table 1: Change in antimicrobial use practices of animal health professionals.*				United Kingdom	Medium
Regional	Powell et al., 2017	Cornwall One Health Antimicrobial Resistance Group.	AHP (+MHP)	AMR, AMS	One Health AMR education at Cornwall Veterinary Association conference. AMU decreased among MHP by 12.8% (in primary care). No data on AHP.	Increased knowledge targeting AHP and MHP. Reduction in human AMU.	One Health approach creating collaboration and understanding.	Limited evaluation of interventions. Limited veterinary involvement.	Cornwall, United Kingdom	Low
Regional	Rees et al., 2021	The Arwain Vet Cymru Project - Veterinary Prescribing Champions (VPC) (incl. webinars, workshops, discussion).	AHP	AMU, AMS	43 veterinarians being VPCs with knowledge about AMS.	Increased AMS knowledge in VPCs with the aim to disseminate this to practices.	Using peers to disseminate knowledge.	Labour intensive for creators and participators. Impact rather than research led.	Wales, United Kingdom	Low
Small Scale	Feyes et al., 2021	AMS programme in veterinary teaching hospital based on the CDC 7 core elements of hospital AMS program.	AHP	AMU, AMR, AMS	Surveillance of AMU and AMR, aim for students to have knowledge of AMS, AMR, and correct AMU (guidelines).	Surveillance data on AMU and AMR. Impact on other aims/outcomes not reported in article.	Targeting AHPs before they start practicing hopefully creating good habits from the start.	Did not measure impact or outcome for most of AMS programme.	Ohio, USA	Low
Small Scale	Lee et al., 2017	Sanitation and education intervention about cleaning milking equipment and udders.	Farmers, cattle	Other	See ***Table 3**: Change in development and/or spread/distribution of AMR.*				Malaysia	Medium
Small Scale	Morgans et al., 2021	Facilitated farmer action groups	Farmers, cattle	AMU, AMR, AMS	Median reduction in HPCIA use was 3.484 mg/kg (p<0.001) Median reduction in General AMU was 0.360 mg/kg (p = 0.719). Qualitive assessment showed increase knowledge.	Statistically significant reduction in HPCIA but not in general AMU. An increased knowledge on AMR, AMU, and AMS.	Generates conversation and understanding not just action.	No control group used. Intervention time consuming (meet every 6 - 8 weeks).	United Kingdom	Medium
Small Scale	Musoke et al., 2020	One Health training - knowledge on AMR and sanitation (case studies, group discussions).	AHP (MHP)	AMS, AMU	See ***Table 2**: Change in uptake and use of AMS by animal health professionals.*				Uganda	Medium
Small scale	Pempek et al., 2022	AMS training for farmers in two parts: didactic presentations, calf-side training, and veterinarian feedback.	Farmers, Cattle	Other	See ***Table 1**: Change in antimicrobial use practices of animal health professionals.*				Ohio, USA	High
Small Scale	Roulette et al., 2017	Knowledge and innovations for: 1) prudent AMU (tape measures & dosage charts (calculate weight for more accurate dosage) and 2) pasteurization of milk (thermometers). Aim was to reduce resistant *E. coli.*	Farmers	AMR	70% of women used their innovations correctly (thermometer), men performed only 18% of dosage steps correctly. Men retained AMR knowledge (0.30) vs. women (0.14).	Two months after innovation some knowledge on AMR was retained and some use of innovation was still present.	Assessed whether knowledge was retained and had impact on action.	Cultural values need to be incorporated in interventions (data from this can be used going forward).	Tanzania	Medium
Small Scale	Sharma et al., 2022	Raising AMR awareness. Two intervention steps performed 1) Focus group and information pack 1 of 4 about AMR, animal health, animal health and AMR or focus group only and (2) Follow up questionnaire.	Farmers, AHP	AMR	Knowledge scores higher amongst farmers participating in intervention meetings (p<0.05) and received intervention 20 (p=0.03) or 3) (p=0.01).	Knowledge score of farmers is higher when given information on animal health or animal health plus AMR compared to those that just participated in meeting or had information on AMR.	Trialling various material vs. no material.	Only post intervention data considered. Focus group findings not considered.	India	Medium
Small Scale	L. Shen et al., 2021	One year of health education-based interventions (training sessions, speakerphone messages, poster, and handbooks) to improve AMU in pigs and humans.	Farmers, swine	AMU	Increase in knowledge around pigs and AMU not statistically significant.	No significant increase in farmer knowledge on AMU in swine.	Repetition within intervention and process evaluation during year.	Only half of farmers at the start of the intervention raised swine through to the end of the intervention.	China	Medium
Small Scale	van Dijk et al., 2017	Participating in AMU reduction policy making.	Farmers, AHP	AMU	See *Table 5: Change in attitudes and perceptions to AMU, AMR, and AMS.*				United Kingdom	Low

AHP, Animal Health Professional;

AMU, Antimicrobial use;

AMR, Antimicrobial resistance;

AMS, Antimicrobial stewardship;

FAO, Food and Agriculture Organisation of the United Nations;

MHP, Medical Health Professional;

Within ‘Secondary outcome’:

AMU, Change in knowledge of appropriate antimicrobial use practices;

AMR, Change in knowledge of AMR (e.g., increased understanding of AMR (microbiological, public health), how AMR spreads and what it effects, the role of farmers/animal health professions in reduction of AMR.

AMS, Knowledge of antimicrobial stewardship.

**Table 5:** Change in attitudes and perceptions to AMU, AMR, and AMS.

Size	Source	Tool/Intervention	Target population	Secondary outcome	Outcome	Impact	Strengths	Limitations	Location	Quality Grade
Small Scale	van Dijk et al., 2017	Participating in AMU reduction policy making.	Farmers, AHP	Role	Farmers and AHP reported thinking more about their AMU (dry cow therapy reduction and cephalosporin reduction), got ideas for moving Herd Health Plans forward.	Reported greater knowledge about AMU and thoughts on their role.	Using those that will be impacted by the policy to create the policy. Feeling more included may give better results.	Lack of a universal way to assess AMU. Limited comparison and overview.	United Kingdom	Low

AMU, Antimicrobial use;

AHP, Animal Health Professional;

Role, Changes in attitudes and/or perception on farmers/animal health profession’s role in reduction of AMR.

**Table 6:** Surveillance strategies.

Size	Source	Tool/Intervention	Target population	Secondary outcome	Outcome	Impact	Strengths	Limitations	Location	Quality Grade
International	Food and Agriculture Organization of the United Nations, n.d.	FAO-PMP-AMR - Help countries to create national action plans (NAPs).		AMU, AMR	Aim to increase awareness, surveillance & research, promote responsible AMU (strengthen governance and allocate resources on country level).	Individual country impact not evaluated.	Working toward unified goal/framework of NAP.	Limited information about tool.		N/A
Continental	EFSA, 2022	Antimicrobial Resistance in zoonotic and indicator bacteria.	Livestock	AMR	Monitoring of zoonotic and indicator bacteria for AMR with data from 27 EU member states (incl. *Salmonella*, *E.coli* (ESBL/AmpC).	Data available for AMR for both humans and animals of zoonotic and indicator bacteria.	Supranational programme, overview of EU - reflection and discussion of each strain.	Does not include clinical break points.	Europe	N/A
Continental	European Medicines Agency, 2022	European Surveillance of Veterinary Antimicrobial Consumption (ESVAC).	AHP, farmers	AMU	Collation of veterinary antibiotic sales in 31 EU countries.	Overview of veterinary antibiotic sales in EU by country and antimicrobial class.	Leverage point and accountability by other EU nations by having overview.	Sales to not equate usage directly.	Europe	N/A
Continental	Mader et al., 2021, 2022	European Antimicrobial Resistance Surveillance network in veterinary medicine (EARS-Vet).	Livestock	AMR	Aim to create surveillance system and collate veterinary clinical AMR isolates from EU countries.	Not yet fully in practice.	Not yet fully in practice.	Not yet fully in practice.	Europe	N/A
National	AURES, 2017	Austrian Report on Antimicrobial Resistance.	Farmer, AHP (MHP)	AMR, AMU	Campylobacter and AMU monitoring for veterinary/food sector (also human sector).	Data on campylobacter and AMU in veterinary sector + data on AMU and other resistance in human.	Joint coordination between human and veterinary.	Limited ability to compare human and veterinary due to different tests and cut offs.	Europe	N/A
National	Bradley et al., 2017	National mastitis control scheme: AHDB Dairy Mastitis Control Plan (DMCP) (includes surveillance and actions).	AHP, Farmers & Cattle	AMU	See ***Table 1**: Change in antimicrobial use practices of animal health professionals.*				United Kingdom	Medium
National	Breen et al., 2017	National mastitis control scheme: AHDB Dairy Mastitis Control Plan (DMCP) (includes surveillance and actions).	AHP, Farmers & Cattle	AMU	See ***Table 1**: Change in antimicrobial use practices of animal health professionals.*				United Kingdom	Medium
National	Cazeau et al., 2022	Resapath - French surveillance network for antimicrobial resistance in bacteria from diseased animals.	Livestock	AMR	AMR from 14 bacteria (incl. *E.coli*, *S. aureus*, *Streptococcus*) monitored across species.	Data on resistance levels from a range of veterinary bacterial pathogens.	Training runs annually to try and align result interpretation at partner labs. Broad/extensive resistance monitoring.	Voluntary submission. Testing run by partner labs.	France	N/A
National	Centers for Disease Control and Prevention, 2015	National Antimicrobial Resistance Monitoring System for Enteric Bacteria (NARMS).	Livestock (retail meat, humans)	AMR	AMR monitoring from bacteria incl. *Salmonella, Shigella, Campylobacter, E. coli O157, Vibrio.*	Data on resistance levels in bacteria incl. *Salmonella, Shigella, Campylobacter, E.* c*oli O157, Vibrio*.	Whole genome sequencing.	Data or reports from 2015 forward available through other sources.	USA	N/A
National	Danish Veterinary and Food Administration, n.d.	VetStat	Farmers, AHP	AMU	Reporting of AMU (and other medication) for food producing animals from farmers, AHPs and pharmacists.	Data available for AMU of food producing animals.	Available on herd level for AHP and farmers to assess own usage.	AMU data not publicly available (through some published through DANMAP).	Denmark	N/A
National	DANMAP, 2022	Danish Integrated Antimicrobial Resistance Monitoring and Research Programme.	Farmers, AHP, livestock (humans)	AMU, AMR	AMR and AMU monitoring.	Data available for AMU and AMR for both humans and animals.	Available and accessible materials and methods section.	AMU reported incl. purchase data, may not reflect species prescription or amount used.	Denmark	N/A
National	Dupont et al., 2017	Yellow card scheme	AHP, farmers, swine	AMU	See ***Table 1**: Change in antimicrobial use practices of animal health professionals.*				Denmark	Medium
National	Federal Office of Consumer Protection and Food Safety, 2020	Federal Office of Consumer Protection and Food Safety, 2020.	Livestock	AMR	Monitoring of zoonoses (incl. *salmonella, L. monocytogenes, E. coli*) and AMR within these in annual reports.	Data available of AMR within zoonoses.	Various stages of production evaluated - allows tracking along food chain.	Not presented alongside human data.	Germany	N/A
National	Federal Office of Public Health FOPH, 2015	Usage of Antibiotics and Occurrence of Antibiotic Resistance in Bacteria from Humans and Animals in Switzerland.	Livestock, AHP, farmers (and human)	AMU, AMR	Monitoring of zoonoses (incl. *salmonella, L. monocytogenes, E. coli*) and AMR within these along with indicator bacteria and veterinary antimicrobial sales in annual reports.	Data available of AMR within zoonoses, indicator bacteria as well as antimicrobial sales.	Sampling from both healthy animals and diagnostic samples.	Sales do does not equate usage. Mg/kg also does not reflect doses (no defined DDD for animals).	Switzerland	N/A
National	Finnish Food Authority, 2022	Finnish Veterinary Antimicrobial Resistance Monitoring and Consumption of Antimicrobial Agents.	Livestock, AHP, farmers	AMU, AMR	Monitoring AMR of zoonoses, indicator bacteria as well as antimicrobial sales in annual reports.	Data on AMR of zoonoses and indicator bacteria & AMU.	Population adjusted sales, mg active ingredient per PCU (mg/PCU).	Narrow resistance testing. Sales does not equate usage. Mg/kg also does not reflect doses (no defined DDD for animals).	Finland	N/A
National	INFAAR, 2020	Laboratory-based surveillance of AMR in fisheries and aquaculture: 1) AMR from healthy fish, and 2) improve AMR awareness in community.	Farmers, aquaculture	AMR	Aim to establish surveillance of AMR in fisheries.	Seemly not yet completed.	Lack of information on the programme.	Lack of information on the programme.	India	N/A
National	JVARM, n.d.	Japanese Veterinary Antimicrobial Resistance Monitoring System.	Farmers, AHP, livestock	AMU, AMR	Monitoring AMU consumption (sales), Resistance in zoonotic and indicator bacteria in healthy animals. Resistance in pathogens in diseased animals.	Data on AMU consumption and resistance in both indicator and zoonotic bacteria as well as isolates from diseased animals.		No data on food product isolate testing as part of surveillance.	Japan	N/A
National	Korean Veterinary Antimicrobial Usage and Resistance Monitoring, 2022	Korean Veterinary Antimicrobial Resistance Monitoring System.	Farmers, AHP, livestock	AMU, AMR	Monitoring AMR in animals and carcass and antimicrobial sales.	Data on AMR and antimicrobial sales available on interactive database.	Unable to find data in English.	Unable to find data in English.	Korea	N/A
National	MARAN, 2021	Monitoring of Antimicrobial Resistance and Antibiotic Usage in Animals in the Netherlands.	Farmers, AHP, Livestock (companion animals)	AMU, AMR	Monitoring AMR of food-borne pathogens, indicator bacteria and Enterobacteriaceae plus antimicrobial sales in annual reports.	Data on AMR from food-borne pathogens, indicator bacteria and Enterobacteriaceae as well as antimicrobial sales.	Dual reporting (however no comparison of isolates).	Limited resistance testing. Sales does not equate usage. Mg/kg also does not reflect doses (no defined DDD for animals).	Netherlands	N/A
National	Simonsen et al, 2022	Usage of Antimicrobial Agents and Occurrence of Antimicrobial Resistance in Norway.	Farmers, AHP, livestock (humans)	AMU, AMR	Monitoring of AMR in zoonotic pathogens, indicator bacteria, clinical isolates, and antimicrobial sales.	Data on AMR from zoonotic pathogens, indicator bacteria and clinical isolates and antimicrobial sales.	Comprehensive dual reporting to same agency.	Limited resistance testing. Sales does not equate usage. Mg/kg also does not reflect doses (no defined DDD for animals).	Norway	N/A
National	Public Health Agency of Sweden and National Veterinary Institute, 2022	SVARM - Sales of antibiotics and occurrence of antibiotic resistance in Sweden.	Farmers, AHP, Livestock	AMU, AMR	Monitoring of AMR in zoonotic pathogens, indicator bacteria, clinical isolates, and antimicrobial sales.	Data on AMR from zoonotic pathogens, indicator bacteria and clinical isolates and antimicrobial sales.	Comparative analysis between human and animal antimicrobial sales & AMR.	Sales to not equate usage directly.	Sweden	N/A
National	Teng et al., 2022	VISAVET Health Surveillance Centre.	Swine	AMR	National AMR surveillance of AMR in food producing pigs through faecal samples at abattoir.	Data on AMR in *Salmonella* from Swine for a 16-year period.	Thorough analysis of specific bacteria, multiple resistance genes and testing methods.	Different antimicrobials tested so data set could not be analysed. MICs and antibiotic susceptibility testing changed over the years. Only high-capacity abattoirs included, does not reflect farms not collaborating with them.	Spain	N/A
National	U.S. Department of Agriculture Animal and Plant Health Inspection Service, 2022	National Animal Health Monitoring System (NAHMS).	Livestock	AMU, AMR	National studies on health and health management of livestock and poultry. Nine studies on AMU, AMS, and AMR (generally *Salmonella, Campylobacter, E. coli*, and *Enterococcus*).	Available national data on AMS, AMR, AMU.	One Health - Integrated report/data presentation for comparison, broader overview of health with AMU.		USA	N/A
National	U.S. Food & Drug Administration, 2022a	The National Antimicrobial Resistance Monitoring System (NARMS).	Livestock (retail meat and human)	AMR	Monitoring system of AMR in enteric bacteria from ill people (CDC), retail meats (FDA) and food animals (USDA).	Data on resistance levels in enteric bacteria.	Integrated data presentation for comparison, partner with Vet-LIRN.	Emphasis on clinical illness isolates only from humans and companion animals (and less on food producing animals.	USA	N/A
National	U.S. Food & Drug Administration, 2022b	Veterinary Laboratory Investigation and Response Network (Vet-LIRN).	Livestock	AMR	Track AMR, create AMS material, and promote AMS within veterinary hospitals.	Available material/AMR educational resources and tracking AMR.	Laboratory network that all test to same standard partner with NARMS.	More emphasis on clinical illness isolates only from humans and companion animals.	USA	N/A
National	Veterinary Medicines Directorate, n.d.	Veterinary Antimicrobial Resistance and Sales Surveillance - UK VARSS.	Farmers, AHP, livestock, swine, poultry, companion animals	AMU, AMR	Monitoring AMR of zoonoses, commensal bacteria of healthy slaughter animals, and clinical AMR surveillance as well as antimicrobial sales and usage in annual reports.	Data available on AMR in both healthy animals and diagnostics as well as antimicrobial sales.	Antimicrobial sales but also AMU reported on electronic medicine books by AHP & farmers.	Some antimicrobials sold to feed mills and exported. AMR within diagnostic samples not representative of entire population.	UK	N/A
Small Scale	Feyes et al., 2021	AMS programme in veterinary teaching hospital based on the CDC 7 core elements of hospital AMS program.	AHP	AMU, AMR	*See **Table 2**: Change in uptake and use of AMS by animal health professionals.*				Ohio, USA	N/A
Small Scale	Ha et al., 2021	Created App for drug stores to report veterinary drug sales in over 3-week period.	Farmer, AHP	AMU	Sales data of veterinary antimicrobials collected from veterinary drug stores using App (on provided tablets) from veterinary drug shops.	Data available for veterinary drug sales from veterinary drug stores.	Ability for government to collect sales data.	3 weeks extrapolated to 1 year - variations in year not accounted for. No indication of compliance levels. Sales does not equate usage. No feed additives counted this way.	Vietnam	Low
Small Scale	Yusuf et al., 2018	iSIKHNAS (Indonesia’s integrated animal health information system).	Farmers, livestock	AMU	Surveillance system for farmers to report medical usage and disease.	Self-reported data available on AMU for livestock farms.	Offers farms a way to track when there is not a national system. Offers data for future research.	Only covers most developed island - not true picture of remote areas. Not true number of animals in which antimicrobial is used.	Indonesia	N/A

AM, Antimicrobial;

AMU, Antimicrobial use;

AMR, Antimicrobial resistance;

AMS, Antimicrobial stewardship;

AHP, Animal Health Professional;

FAO, Food and Agriculture Organization of the United Nations;

MHP, Medical health professional;

Within ‘Secondary outcome’:

AMU, Surveillance of AMU/AM sales;

AMR, Surveillance of AMR.

**Table 7:** Other.

Size	Source	Tool/Intervention	Target population	Secondary outcome	Outcome	Impact	Strengths	Limitations	Country	Quality Grade
International	Food and Agriculture Organization of the United Nations, 2020	FAO Assessment Tool for Laboratories and AMR Surveillance Systems (FAO-ATLASS).		Review of Lab and AMR surveillance systems	28 countries have had assessments performed.	Defined targets for national AMR surveillance in food and agriculture sectors.	Availability of a working tool.	Limited information about tool.		N/A
International	World Organisation for Animal Health, 2019	OIE-Performance of Veterinary Services (PVS) Pathway.		Assessment of animal health situation, incl. AMR	Self-reported review/score of animal health in country.	Tool/score for countries to work towards improving.	Standardised scoring system for all countries.	Self-reporting requires countries to choose to submit. Risk of participation bias as only countries with resources and interest might participate.		N/A
International	WHO, 2022	Tripartite AMR country self-assessment survey (TrACSS).		AMR Monitoring and Surveillance network	Aim for countries to review progress in implementing actions to address AMR at the national level, and to report annually at the global level.	Standardised progress reports for countries to address AMR.	Overview of nations’ effort, evaluate own efforts, see other countries efforts, multisectoral.	Self-reporting requires countries to choose to submit. Risk of participation bias as only countries with resources and interest might participate.		N/A
International	World Organisation for Animal Health, n.d.	Veterinary Legislation Support Program (VLSP).		Assessment of veterinary legislation	Report for 137 countries on veterinary legislation that is aimed at creating legislation that reduces biological threat and AMR.	Knowledge about gaps and weaknesses of veterinary legislation, including those pertaining to AMR.	Knowledge sharing about legislation.	Report/review does not equal action/change.		N/A
International	Food and Agriculture Organization of the United Nations, n.d.	Tool for a Situation Analysis of AMR risks in the Food and Agriculture Sectors on a national level.		Report on AMR risk and improvements	Aim to provide picture of current situation and guide decisions.	Individual report impact not evaluated on.	Added support for countries with less resources so they do not have to make their own evaluation.	Only as useful as the country implementing. No assessment if action plans are suitable for country.		N/A
Small Scale	van Dijk et al., 2017	Participating in AMU reduction policy making	Farmers, AHP	Policy created	*See: **Table 5**: Change in attitudes and perceptions to AMU, AMR, and AMS.*				United Kingdom	Low

AMU, Antimicrobial use;

AMR, Antimicrobial resistance;

AMS, Antimicrobial stewardship;

AHP, Animal Health Professional;

FAO, Food and Agriculture Organization of the United Nations.

Throughout the key findings described in the sub-sections below, studies were highlighted as examples to illustrate the main themes of the outcomes. Further information on all the included studies can be found in **Tables 1**–**7**.

### 3.1 Change in AMU practices of AHPs and farmers

Change in AMU practices of AHPs was reported in 22 interventions across 24 studies (**Table 1**). The most frequent aspects measured were reduction of volume/weight of AMU at herd level (12/22), reduction in the volume of HPCIAs used (9/22), reduction of volume of AMU for a specific diagnosis (5/22), and reduction of volume of AMU for livestock animals at regional, national, or continental level (5/22). Nine of the twenty-two (9/22) interventions that assessed change in AMU practices were aimed at AHPs and 20/22 were aimed at farmers. Most of the interventions in this category were implemented in Europe (15/22), followed by North America (5/22) and Asia (2/22). The geographical coverage of the interventions varied from continental (1/22), national (3/22), and regional (1/22) to small scale (17/22). The quality of the intervention studies design was considered high (7/24) and medium (17/24).

The following subsections further describe the interventions in which change in AMU practices was assessed, and the related impacts, limitations, and gaps.

#### 3.1.1 Reductions in AMU at herd level

Herd-specific interventions showed a reduction in AMU. These interventions focussed on implementation of farmers and AHP education, increased health and welfare care (e.g., stable climate, management), biosecurity (external and/or internal), and vaccine strategy (Collineau et al., 2017; Speksnijder et al., 2017; Raasch et al., 2020; Gerber et al., 2021; Gomez et al., 2021; Phu et al., 2021; Pempek et al., 2022; Schreuder et al., 2022). The targeted study groups included cattle farmers, swine farmers, broiler farmers, and AHPs (Collineau et al., 2017; Speksnijder et al., 2017; Raasch et al., 2020; Gerber et al., 2021; Gomez et al., 2021; Phu et al., 2021; Pempek et al., 2022; Schreuder et al., 2022).

As a first example of herd-specific interventions, Raasch et al. (2020) measured the reduction of AMU and HPCIAs among a swine farmer population in Belgium, Germany, France, and Sweden. A significant median reduction of AMU of 35% was reported. After the intervention was implemented, the duration for which pigs were treated reduced from 25% of their expected lifespan (200 days) to 16% (Raasch et al., 2020). The authors reported a compliance rate of 93% with the intervention plans by the target population. The strengths of this intervention were customised interventions for each herd and a broken-down assessment of AMU by diagnosis and age group. However, no control group was used on the basis that this group could change over the year (Raasch et al., 2020). This means it was not possible to adjust results for external factors that otherwise might be seen in a control group.

In a second example, reduction in AMU at the herd level was assessed for cattle farmers in Ohio, USA. A 50% reduction in AMU for calves was accomplished through didactic presentations, calf-side training, and veterinarian feedback for farmers. There was also an increased understanding of AMS and higher correct identification of cases in need of AMU (50%, 73/146) vs. the control group (14.3%, 9/63) (p=0.002) (Rojo-Gimeno et al., 2016; Pempek et al., 2022). This intervention allowed for an integrated approach looking at both AMU but also testing farmer knowledge and not relying on self-reporting. The observed weaknesses in that study were that the control and test groups were not randomly allocated, and both were presumed to have an increased interest in AMR, potentially biasing the outcomes. There was also no follow-up post-intervention measurement performed to evaluate if improvement was ongoing, and this was made difficult by veterinarians being reluctant to provide information on curative antimicrobials.

In the final example, Gerber et al. (2021) measured general AMU, diagnosis-specific usage, and HPCIAs usage amongst cattle farmers in Switzerland. Farmers picked the interventions to implement in their herds/farms from a pre-defined list of 17 udder, uterine, or calf health interventions. Udder or uterine health strategies resulted in a reduction in AMU (p < 0.04). Calf health interventions did not result in reduction in AMU. Allowing the farmers to choose herd-specific interventions from a pre-defined list allowed farmers to have partial autonomy. Observed weaknesses were the test and control groups were of different herd-sizes, breeds, and milk yields, which made comparison and interpretation of outcomes challenging. In addition, no information was collected on why the farmers chose their specific interventions (Gerber et al., 2021).

The financial benefit of AMU reduction was only explored in one intervention in two papers (Rojo-Gimeno et al., 2016; Postma et al., 2017). Increased net profit was recorded for a broad intervention that included herd management, biosecurity, and vaccination strategy customised to age groups of swine. At the same time, a decrease in treatment incidence of 52% and 32%, from birth to slaughter and for breeding animals, respectively, was reported.

#### 3.1.2 Interventions reporting reduction in HPCIAs

Reduction in the use of HPCIAs (n = 9/22) was reported as part of broader interventions (Kuipers et al., 2016; Postma et al., 2017; Gerber et al., 2021; Morgans et al., 2021; Moura et al., 2022). Postma et al. (2017) noted a reduction of long-acting ceftiofur in sucklers by 83% and Gerber et al. (2021) reported a reduction in HPCIAs for treatment of udder related ailments (p = 0.05). However, reduction in use of HPCIAs was not always coupled with a general AMU reduction. An intervention targeting cattle-farmer-facilitated action groups assessed both general AMU and use of HPCIAs in cattle farmers in the United Kingdom and reported a reduction in use of HPCIAs of 3.484 mg/kg (p<0.001) but an overall median AMU reduction of 0.360 mg/kg (p = 0.719) (Morgans et al., 2021). In the same study, participant knowledge about AMR, AMS, and AMU at pre and post intervention was assessed qualitatively and an increase in the measured outcomes was reported. The noted study limitations were the lack of a control group and a societal push for AMR awareness at the time (Morgans et al., 2021).

#### 3.1.3 Reduction of volume of AMU for a specific diagnosis

Five interventions (5/22) were implemented at the regional, national, or continental level and all five addressed a specific diagnosis (5/20) (**Table 1**). Four of the 5 interventions were at the regional, national, or continental level and measured the following: AMU in European farmers and AHPs (The European Parliament and The Council of the European Union, n.d)., reduction in ceftiofur use by AHPs and poultry farmers in Canada (Agunos et al., 2017; Agunos et al., 2018), AMU and HPCIAs reduction in AHPs and swine farmers in Denmark (Jensen et al., 2014; Dupont et al., 2017), and general AMU, AMR, diagnostic specific AMU reduction in dairy cattle farmers in the United Kingdom (Bradley et al., 2017; Breen et al., 2017), and HPCIAs reduction in dairy cattle farmers and AHPs in the Netherlands (Moura et al., 2022).

A fifth intervention, ‘Yellow Card System’, was at the national level and was aimed at reducing AMU and HPCIAs use in swine farmers and AHPs in Denmark (Jensen et al., 2014; Dupont et al., 2017). The intervention required swine farms to reduce their AMU to pre-set levels and resulted in 38.4 – 56.2% reduction of mg active ingredients/pig/day with increased use of vaccines, and decreased herd medication was reported as the biggest perceived influencing factor (Jensen et al., 2014; Dupont et al., 2017). However, these factors were only assessed in herds with > 10% reduction in AMU and self-reported by farmers and AHPs. This study included a large national sample size and excluded herds with < 10% reduction in AMU. Other national interventions overlapped with diagnostic-specific interventions. A national mastitis control scheme assessed AMU by AHPs and dairy cattle farmers in the United Kingdom (Bradley et al., 2017; Breen et al., 2017). This intervention resulted in a 40% reduction in use of intramammary medication in lactating cows and a 20% reduction in clinic mastitis rates, achieved through AHP and farmer training. However, this intervention was only noted in a letter and conference proceedings and no information was provided on strengths and limitations (Bradley et al., 2017; Breen et al., 2017).

#### 3.1.4 Summary of change in AMU practices of AHPs and farmers

This category of interventions primarily focused on farmers and used herd-specific interventions to reduce AMU with success reported for both overall AMU reduction and reduction in use of HPCIAs. One study required farmers to select interventions from a pre-set list (Gerber et al., 2021). There was overlap of the primary outcomes measured across interventions and many studies also featured other primary outcome measures. Diagnosis-specific interventions were aimed at changing AMU in cases of mastitis and calf diarrhoea (Bradley et al., 2017; Gomez et al., 2017; Bailey et al., 2019). Studies involved pre- and post-intervention measurement of outcomes. Only a few studies reported use of control groups to account for other external influences (Kuipers et al., 2016; Becker et al., 2020).

### 3.2 Change in uptake and use of AMS by AHPs and farmers

Within this primary outcome, change in prescribing habits (n=4/8) was the most frequently measured, followed by increased adherence to guidelines (n=2/8) and increased use of diagnostics (n=2/8) (**Table 2**). There were additional aspects from ‘Other’ category of interventions reported within this primary outcome (n=6/8). Farmers (6/8) and AHPs (5/8) were almost equally targeted. Four interventions were implemented in Europe (4/8), two in Africa (2/8), and two in North America (2/8). The interventions were distributed across the levels: continental (1/8), national (1/8), regional (2/8), and small-scale (4/8). Of these interventions, two (2/8) were legislative interventions. The quality of intervention design ranged from high (1/7), medium (4/7), to low (2/7). The interventions, impact, outcome, and limitations are described in **Table 2**.

#### 3.2.1 Change in prescribing habits

The studies reporting change in prescribing habits focussed on herd health plans and educational interventions. As an example, a study in Uganda used a One Health approach and focused on change in prescribing, guideline use, and diagnostic use in medical, healthcare, and AHPs (Musoke et al., 2020). Medical health professionals self-reported improved handwashing (57.3%), guideline use (52.9%), treatment based on diagnostics (44.1%), and reduction in unnecessary AMU (51.3%). Participation of AHPs was low compared to medical health professionals (Musoke et al., 2020). A disadvantage of self-reporting is perception may not translate to actual action; just because the participant says they are doing something, it does not mean they are. The other interventions surrounding prescribing habits, including the intervention of prescribing champions and herd health plans, are discussed in other sections (Raasch et al., 2020; Rees et al., 2021; Pempek et al., 2022).

#### 3.2.2 Change of AMS through legislation

Change in AMS through legislation was reported. Two examples are the ‘California State Bill 27’ aimed at farmers in California, USA (Abdelfattah et al., 2022), and Regulation (EU) 2019/6 on veterinary medicinal products and repealing Directive 2001/82/EC aimed at both farmers and AHPs in Europe (The European Parliament and The Council of the European Union, n.d). The ‘California State Bill 27’ states that usage of antimicrobials of medical importance for humans for livestock requires a prescription. Assessment of this intervention indicated self-reported change in disease management including increased use of diagnostics (29.4%) and an increased use of alternatives to antimicrobials (26.8%) (Abdelfattah et al., 2022). This study was limited by a low response rate and possible response bias. As mentioned previously, self-reporting change may not translate to action. There was no report on AMU suggesting it was not evident whether self-reported change resulted in action (Abdelfattah et al., 2022). The latter, EU regulation, bans medication through feed or to groups for livestock use (The European Parliament and The Council of the European Union, n.d.). No data was presented within the legislation about the effect of this legislation (The European Parliament and The Council of the European Union, n.d.). In general, there were few studies evaluating legislation/bills.

#### 3.2.3 Other reported aspects

Other aspects were reported under the primary outcome measure, change in uptake and use of AMS by AHPs and farmers. The first aspect was improving sanitation (i.e., improving hand washing and biosecurity) in both AHPs in Uganda and California (Musoke et al., 2020; Abdelfattah et al., 2022). The second aspect was improving dosage accuracy in cattle farmers in Ohio, USA (Pempek et al., 2022). These interventions were described in earlier sections and under the primary outcome measure ‘change in AMU of AHPs.’

#### 3.2.4 Summary of change in uptake and use of AMS by AHPs and farmers

The interventions reported under this category illustrated how both voluntary programmes and legislation can create an impact on AMS. However, it is important to note the impact of many of these interventions were self-reported (Musoke et al., 2020; Abdelfattah et al., 2022). This carries the risk of response bias. Social and moral responsibility perceived by the reporting individuals may therefore influence the responses (Bradburn et al., 2004).

### 3.3 Change in development and/or spread of AMR

Change in development and/or spread of AMR was reported. The most frequent aspect measured was the reduced frequency of resistance genes within detected strains (21/30) followed by reduced frequency of bacterial strains (9/30), reduced frequency of resistance genes detected/isolated in food products (meat, milk, egg, etc) (5/30), and reduced frequency of resistance genes detected/isolated within the herd environment (3/30) (**Table 3**). The interventions were conducted in Europe (12/30), North America (10/30), Asia (4/30), South America (3/30), and Africa (1/30). The interventions were primarily small-scale (28/30) with two interventions conducted on a national level (2/30). The quality of study design was split between high (17/30) and medium (13/30) (**Table 3**).

#### 3.3.1 Reduction in resistance strains

Reduced frequency of resistance genes within bacterial strains (19/26) was reported primarily at a small-scale level (17/19) and twice at a national level (2/19). Findings from the interventions at the national level are presented separately from the small-scale interventions.

##### 3.3.1.1 National projects

Two interventions conducted at a national level focused primarily on ceftiofur resistance in broilers. The first intervention reported a voluntary reduction of ceftiofur and assessed the reduction of resistant strains in Japanese hatcheries (Hiki et al., 2015). Testing was performed on one faecal sample per farm with commercially available kits and to country standards. This longitudinal study did not have a control group or assess confounding but evaluated multiple resistance genes (Hiki et al., 2015). The second intervention, the Canadian Integrated Program for Antimicrobial Resistance Surveillance, tested for ceftiofur and other resistance genes in farms, abattoirs, and retail products (Agunos et al., 2017). All isolates were tested at national reference laboratories for continuity and to allow for comparison of results (Agunos et al., 2017). Neither intervention performed assessments of mortality or animal health. Both interventions used different reduction strategies and testing methods, but both reported reduced ceftiofur resistance in broiler production.

##### 3.3.1.2 Small scale

**Figure 4:**
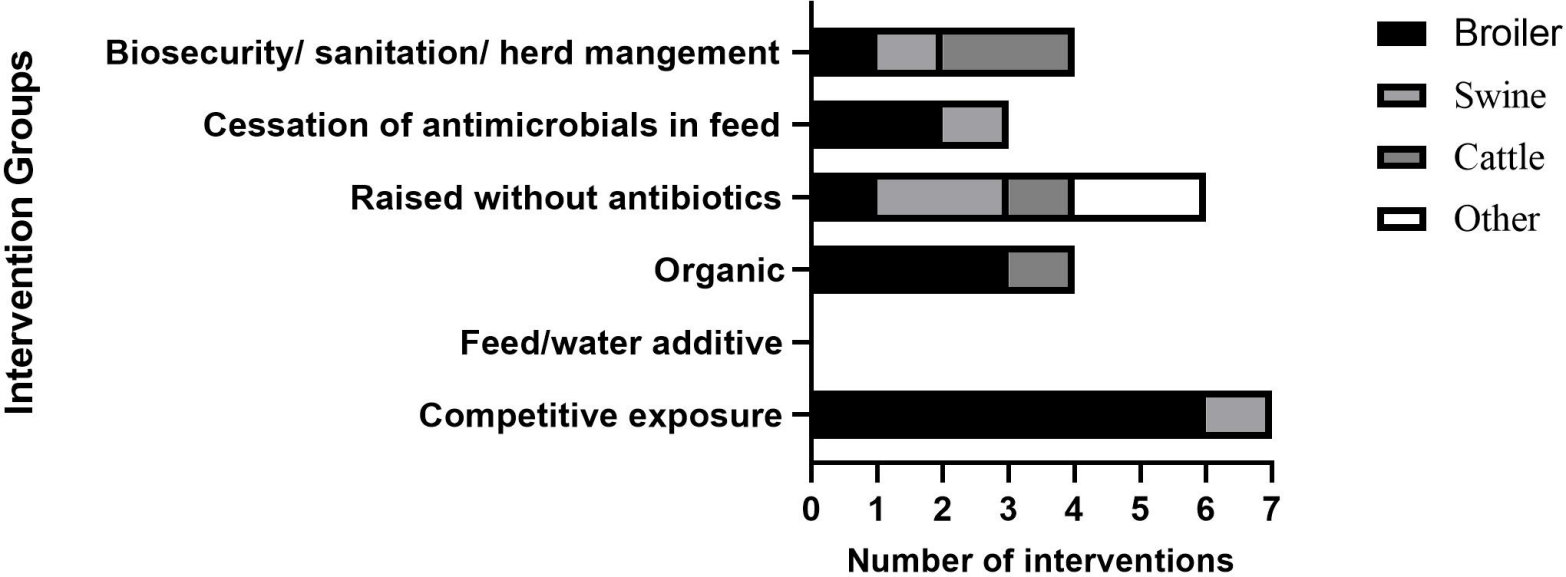
Distribution of interventions that assessed change in development of AMR with a focus on reducing AMR resistance genes. ‘Other’ in RWA accounts for intervention that includes broilers, swine, and cattle, and meat products.

Small-scale interventions were also reported and mainly focused on resistant strains in broilers (14/21), along with swine (7/21), cattle (5/21), goats (1/21), and a range of end meat products (1/21). The interventions assessed a range of parameters including biosecurity/sanitation (4/21) (Hao et al., 2013; Dorado-García et al., 2015a; Dorado-García et al., 2015b; Wanninger et al., 2016; Brusa et al., 2019), animals raised without antibiotics (6/21) (Pedroso et al., 2013; Wanninger et al., 2016; Thapaliya et al., 2017; Vikram et al., 2017; Haskell et al., 2018; Loayza-Villa et al., 2021), cessation of antimicrobials in feed (3/21) (Chinwe et al., 2014; Verrette et al., 2019; Shen et al., 2020) and competitive exposure (7/21) (Nuotio et al., 2013; Pedroso et al., 2013; Ceccarelli et al., 2017; Mourand et al., 2017; Methner et al., 2019; Dame-Korevaar et al., 2020a; Dame-Korevaar et al., 2020b). The distribution of the various interventions is summarised in **Figure 4**.

Competitive exposure was one of the interventions used in broilers and included use of commercial products, pre-and probiotics, as well as specifically created bacterial compositions with positive effects (Nuotio et al., 2013; Pedroso et al., 2013; Ceccarelli et al., 2017; Mourand et al., 2017; Methner et al., 2019; Dame-Korevaar et al., 2020a; Dame-Korevaar et al., 2020b). Two specific examples of interventions involving competitive exposure were the use of unselected fermented intestinal bacterial and/or a selection of pre- and pro/biotics in broilers in the Netherlands (Dame-Korevaar et al., 2020a), and use of a commercial natural live intestinal flora, Aviguard, to target ESBL-*E.coli* in broilers in The Netherlands (Dame-Korevaar et al., 2020b). The former intervention had no effect when unselected fermented intestinal bacterial and a selection of pre- and pro/biotics were given on the same day (Day 0) as the challenge ESBL *E.coli* (Dame-Korevaar et al., 2020a). A reduced excretion of CTX-M-1- *E.coli* was seen when the challenge was given on day 5 after unselected fermented intestinal bacterial and a selection of pre- and pro/biotics. The study was limited by the short time frame (5 days) and experimental conditions. There is a gap in information on whether reduction in resistance is linked to disease or end meat contamination (Dame-Korevaar et al., 2020a).

In the latter study, Aviguard was administered to chicks right after hatching and challenged with CTX-M-1-*E.coli* on day 5 (Dame-Korevaar et al., 2020b). Of the test group, 0/200 broilers were CTX-M-1-*E.coli* positive on day 21 vs. the control with 187/200 positive. Multiple scenarios were tested and CTX-M-1-*E.coli* swabbing occurred every day. A potential limitation of this study was performance in semi-field conditions under stringent biosecurity means results may not translate to field conditions (Dame-Korevaar et al., 2020b). Like the previous report, disease and end meat levels were not assessed. This study suggested competitive exposure was successful within certain criteria such as high biosecurity and short time frames, but more knowledge is needed on the effect of longer timeframes and mechanism of human transmission.

A reduction in resistance levels following herd management and sanitation interventions in livestock was reported. A study in the Netherlands reported a reduction of 31 MRSA-positive herds to 29, and a 44% reduction in AMU, defined daily dosages animal per year, in swine (Dorado-García et al., 2015a). This was achieved by improving personnel and farm hygiene as well as changing animal contact structure. Having separate water pipes from medication pipes, specific rooms for deliveries, and designated sow groups, were all positively correlated with reducing MRSA. A limited intervention period (18 months) and pooling of samples may however lead to inaccuracies in measurement of outcomes (Dorado-García et al., 2015a).

#### 3.3.2 Reduction in bacterial strains

**Figure 5:**
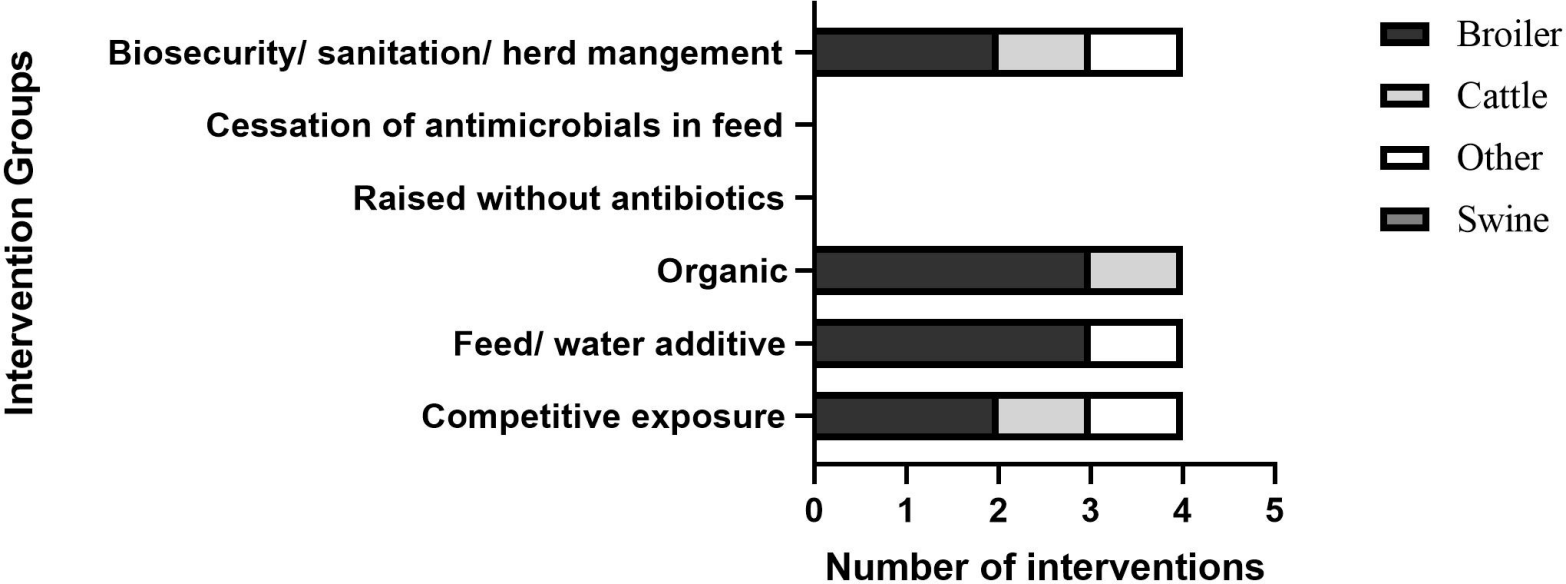
Distribution of interventions that assessed change in development of AMR with a focus on reducing bacterial strains. ‘Other’ in ‘biosecurity/sanitation/herd management’ and ‘feed/water additive’ is same intervention.

The range of interventions focused on reducing bacterial strains (**Figure 5**) was similar to those for resistant strains (**Figure 4**). Broilers were the most frequently targeted animal group (5/9). Others were cattle (1/9) and goats (1/9). Unlike interventions focused on resistance genes, there was a larger emphasis on feed/water additives (4/9) (Hao et al., 2013; Rubio-García et al., 2015; Wanninger et al., 2016; Kannan et al., 2019), on cessation of antimicrobials (1/9) (Chinwe et al., 2014), and biosecurity/sanitation (3/9) (Bahrndorff et al., 2013; Luyckx et al., 2015; Lee et al., 2017; Kannan et al., 2019) (**Figure 5**).

Two examples of feed and drink additives used in the interventions were dietary brown seaweed used to reduce rumen *E.coli* in goats (Kannan et al., 2019) and an allostatic modulator in drinking water and feed withdrawal from broilers to reduce coliforms (Rubio-García et al., 2015). The first intervention investigated microbiological contamination of goat carcasses in Georgia, USA (Kannan et al., 2019). To determine the effect of brown seaweed and chlorinated wash on microbiological contamination of carcasses, bucks were fed seaweed as a supplement and the feed was sprayed with 50 mg/L chlorinated water. Rumen but not skin *E.coli* count was reduced following feeding with seaweed (p <0.05). Skin count was reduced after chlorinated wash (p < 0.05) (Kannan et al., 2019). No information was provided on the contamination of meat in a production (abattoir) setting or transmission to humans. The second intervention aimed to reduce coliforms in broilers and end meat in Mexico (Rubio-García et al., 2015). Broilers were given an allostatic modulator in tap drinking water and a ten- or sixteen-hour feed withdrawal before slaughter shipment. The allostatic modulator contained electrolytes, acetylsalicylic acid, and ascorbic acid. Allostatic modulators aim to reduce allostasis (chronic stress). Reduction in coliforms (p = 0.014) and total aerobic mesophilic bacteria (p = 0.0001) were reported and the intervention was considered financially reasonable and accessible. A limitation of this study was it was performed under experimental conditions only (Rubio-García et al., 2015).

Two interventions on biosecurity and sanitation were reported and these included implementation of education and cleaning protocols. In the first intervention conducted in Belgium, the reduction of bacterial strains detected in broilers was assessed through on-farm cleaning protocols used by farmers (Luyckx et al., 2015). Sanitation of the broiler houses with commercial products containing sodium hydroxide resulted in 86% reduction in the number of *E. coli*-positive swabs (1-3% difference depending on soaking and water temperature) (Luyckx et al., 2015). The second intervention investigated the reduction in bacterial strains in fresh milk samples of cattle in Malaysia (Lee et al., 2017). The intervention was education of farmers on udder and machine sanitation and resulted in a 40% log reduction of *Staphylococcus aureus* in fresh milk samples (Lee et al., 2017). A limitation of this study was that statistical significance was not reported and a clear description of how the training was performed was not provided.

#### 3.3.3 AMR in the environment and food

Articles reported on interventions focussed on the environment (n = 3) and food products (n = 4). Three studies focused on the environment and area around herds. The first study investigated bacterial strains in broilers and the environment in China (Hao et al., 2013). This intervention focused on sanitation, specifically the use of acidic water (pH 5.0 – 6.0) wash containing chlorine to reduce *Salmonella* spp. and *E.coli* in broiler houses and resulted in a 16% reduction in *Salmonella* spp and *E.coli* in broiler houses (Hao et al., 2013). A limitation of this study was the intervention was not applicable to bird housing with cages. The second intervention focused on both sanitation and reducing AMU and investigated resistant bacterial strains in veal cattle in the Netherlands (Dorado-García et al., 2015b). The intervention reported that cleaning and disinfecting negatively impacted the MRSA burden in the environment around veal cattle (Dorado-García et al., 2015b). This intervention was implemented for two production cycles with different techniques and under short time frames (12 weeks) making comparison of results difficult. A third study investigated bacterial strains on swine farms, in the surrounding farm environment, and in meat products in China (Shen et al., 2020). There were significant reductions in MCR-1-positive *E. coli* after the cessation of colistin as a feed additive. This was both at farm level (p < 0.0001), in food (pork) (p < 0.0001), and in the environment (soil and water around slaughterhouses) (p < 0.0001).

#### 3.3.4 Summary of change in development and/or spread of AMR

The design of interventions under this category varied. Some interventions measured outcomes at pre and post intervention to assess change in outcomes, whereas other interventions used control herds. Experimental studies were used to evaluate outcomes within this category, more than for any other primary outcome measurement. Findings from experimental studies do not necessarily translate to or are feasible in field conditions. Replicating these findings in field conditions is an important next step to assess if the interventions work in the real-world situations. Some of the reported interventions run for a short time frame and no indication of disease level, transmission to humans, or end meat contamination was assessed (Dame-Korevaar et al., 2020a; Dame-Korevaar et al., 2020b; Shen et al., 2020).

### 3.4 Change in knowledge of appropriate AMU practices, AMR, and AMS

**Figure 6:**
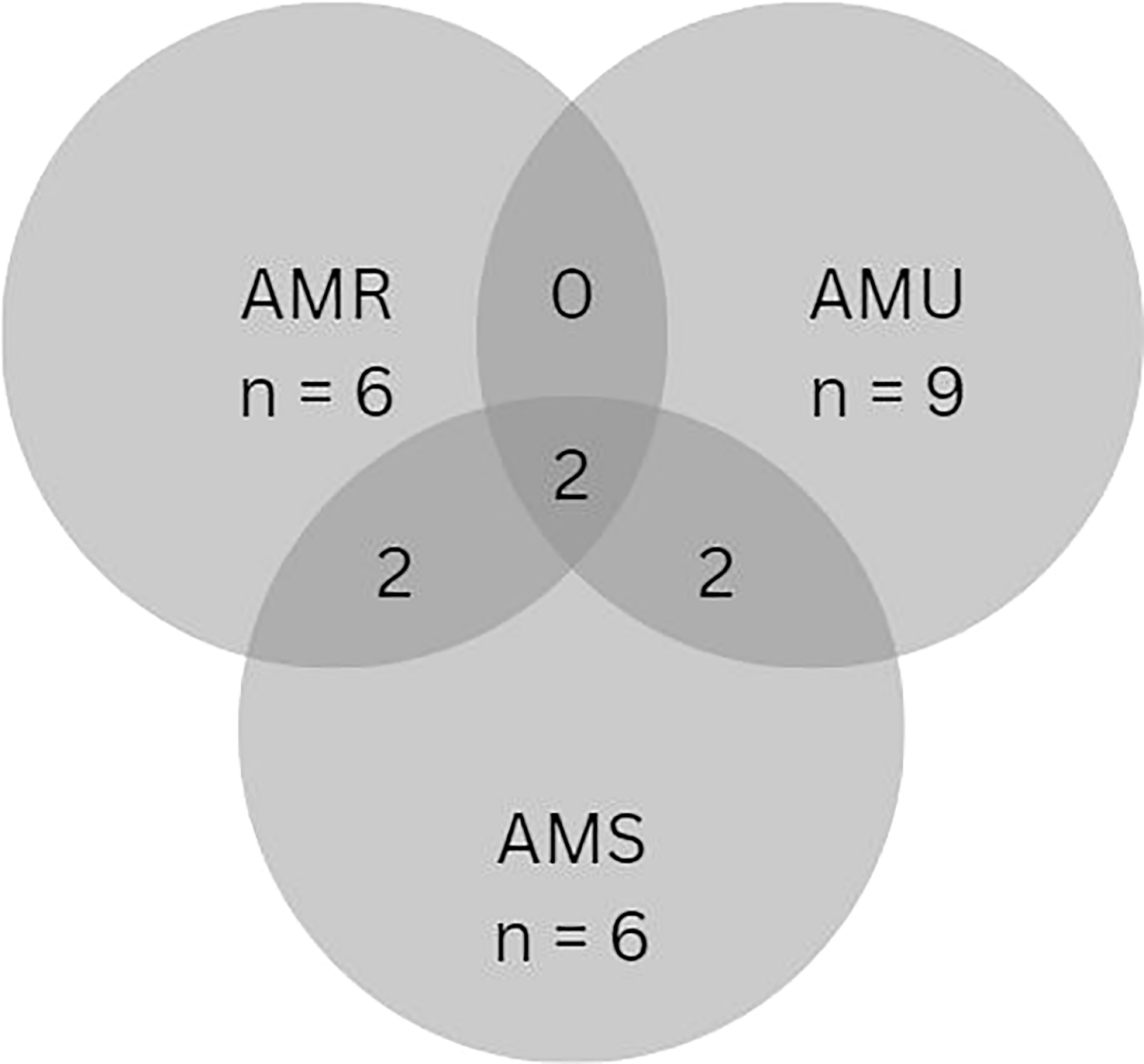
Distribution of interventions that assessed change in knowledge with a focus on AMU practices, AMR, and AMS within secondary outcome measurements.

There were 14 reported interventions within the primary outcome, change in knowledge of appropriate AMU practices, AMR, and AMS. These included change in knowledge of appropriate AMU practices (n = 9), change in knowledge of AMR (n = 6), and change in knowledge of AMS (n = 6), with overlapping observed within the interventions (**Figure 6**). There were also interventions with aspects that did not fit within the predefined groupings (3/14). AHPs and farmers were targeted in 6/14 and 8/14 of the interventions. The interventions were conducted in high income countries in Europe (5/14) and North America (2/14) and less in LMICs within Africa (2/14), and Asia (3/14). Interventions featured across all levels; international (2/14), national (1/14), regional (2/14), and small scale (9/14) (**Table 4**). A description of interventions in high income countries and LMICs is provided below. The quality of design of these studies were scored as medium (7/12), low (4/12) and high (1/12).

#### 3.4.1 Change in knowledge in high income countries

Interventions in this primary outcome measure were mostly in high income countries (Bradley et al., 2017; Powell et al., 2017; van Dijk et al., 2017; Feyes et al., 2021; Morgans et al., 2021; Rees et al., 2021) and overlapped with other primary outcomes. For example, the use of farmer-facilitated groups reduced AMU while increasing knowledge around AMU, AMR, and AMS for dairy farmers in the United Kingdom (Morgans et al., 2021). This intervention was considered time-consuming as required meetings every 6 – 8 weeks but allowed for conversation and discussion to create understanding (Morgans et al., 2021). In another study, the Arwain Vet Cymru Project created veterinary prescribing champions with the aim of changing the behaviour of AHPs in Wales and increasing knowledge of AMU and AMS through webinars, workshops, and discussions (Rees et al., 2021). The impact and limitations of the dissemination were not reported. This intervention was reported to be labour-intensive for the creators and participants (Rees et al., 2021). In another intervention, increased knowledge of farmers and reduction in AMU in calves in Ohio, USA was achieved through didactic presentations and calf-side training (Pempek et al., 2022). Limitations were not reported regarding knowledge acquisition, but other limitations were reported as noted earlier in the section on change in AMU practices of AHPs.

#### 3.4.2 Change in knowledge in LMICs

Interventions focussed on knowledge acquisition were also conducted in LMICs (Roulette et al., 2017; Musoke et al., 2020; Shen et al., 2021; Sharma et al., 2022). While knowledge acquisition was part of a broad intervention in high income countries, this was conducted as a single activity in LMICs. Two interventions that focused on assessing knowledge about AMR were reported in LMICs. The first intervention assess knowledge on AMR and animal health among farmers and AHPs in India (Sharma et al., 2022). The target participants attended meetings and were given ‘knowledge packs’ on AMR and/or animal health, to raise AMR awareness. Higher knowledge scores were reported for farmers that participated in the meetings (p<0.05) and received information on animal health (p=0.03) or animal health and AMR (p=0.01). A key limitation of this study was it did not include translation of knowledge to actions or compare a pre-post intervention knowledge score (Sharma et al., 2022). In the second intervention, AMR was assessed after education on AMR was given to farmers in Tanzanian Masai communities (Roulette et al., 2017). Additionally, tape measures and antimicrobial dosage charts were given to men, and women received thermometers for milk pasteurization. At a 2-month follow-up, men retained more AMR knowledge (30%) compared to women (14%). However, 70% of women used their innovations correctly (thermometer) whereas men only performed 18% of dosage steps correctly (Roulette et al., 2017). A strength of this study was that cultural aspects and gender roles were taken into consideration. A limitation of this study was knowledge retention about AMR and innovation use were not evaluated as potential influences of each other. In general, there was limited information on interventions focussed on change in knowledge in LMICs and no demonstrated evidence of knowledge translating to action.

#### 3.4.3 A summary of the change in knowledge of appropriate AMU practices, AMR, and AMS

The reported interventions illustrate that knowledge on AMR can be learned and retained (Roulette et al., 2017; Sharma et al., 2022) and assessed using pre- and post-intervention testing. However, there is need to gain more understanding of whether knowledge provided to farmers and AHPs translates to action and if there is sustainable change. The intervention conducted by Morgans et al. (2021) aimed to create sustained change through peer-to-peer learning and prescribing champions. However, there was no measurement of outcomes. Interventions evaluating outcome measurements are needed to understand the impact of these interventions.

### 3.5 Change in attitudes and perceptions to AMU, AMR, and AMS

Only one article investigated the change in attitudes and perceptions (n = 1) and did so as a small-scale qualitative assessment of farmers and AHPs in the United Kingdom (van Dijk et al., 2017) (**Table 5**). After participating in the creation of an AMU reduction policy, farmers and AHPs provided thoughts on their AMU practices and how these could be incorporated into their herd health plans. The responses were individual statements on an *ad hoc* basis (van Dijk et al., 2017). This study suggests that using stakeholders (such as AHPs and farmers) that will directly be impacted by policy to create policy could result in the stakeholders feeling more included and motivated potentially resulting in better policy outcomes.

### 3.6 Surveillance strategies

Interventions involving surveillance strategies (30/90) were primarily conducted on a continental (3/30) and national (23/30) level. Most of the surveillance strategies focused on both AMU and AMR (12/30), some focused solely on AMR (9/30), or AMU (4/30). Of those focussing on both AMU and AMR, some considered a One Health approach and provided human data (6/12). The largest number of surveillance strategies were reported in Europe (17/30) and less in Asia (5/30) and North America (5/30). No surveillance strategies were reported in Africa or South America. A detailed description of surveillance involving a One Health approach or focussed on AMU and AMR is provided below. Small-scale interventions (3/30) are presented separately.

#### 3.6.1 One Health-focused strategies

Within national surveillance activities involving AMR and AMU, there was a focus on One Health. The most comprehensive surveillance strategies included AMR in zoonotic pathogens, indicator bacteria, and clinical isolates for both humans and animals along with antimicrobial sales in annual reports. These strategies were most reported in Europe and Asia. Half (6/12) of the reported strategies provided a comparison of human and veterinary isolates (JVARM, ; Federal Office of Public Health FOPH, 2015; MARAN, 2021; DANMAP, 2022; Korean Veterinary Antimicrobial Usage and Resistance Monitoring, 2022; Simonsen et al., 2022).

#### 3.6.2 AMU-focused strategies

In other countries that did not report AMU and AMR together, surveillance strategies were split, or reported aspects on AMU or AMR or human and veterinary isolates, separately. Surveillance strategies focusing on AMU used medical sales data. As an example, on a continental level, the European Surveillance of Veterinary Antimicrobial Consumption (ESVAC) collates antimicrobial sales data from 31 European Union (EU) countries, offering an overview and accountability for usage (European Medicines Agency, 2022). These exist at country level in Europe (Danish Veterinary and Food Administration, n.d.; Finnish Food Authority, 2022).

#### 3.6.3 AMR-focused strategies

Surveillance of AMR on its own exists in multiple forms, on both a national and continental level primarily in Europe and North America. On a continental level, the EU collates AMR data through The European Food Safety Authority (EFSA). Monitoring is conducted for zoonotic and indicator bacteria for AMR (incl. *Salmonella* spp., *E.coli* (Extended Spectrum Beta-Lactamase (ESBL)/AmpC beta-lactamases (AmpC)) with data from both human and livestock isolates from 27 EU member states (EFSA, 2022). Under creation is the European Antimicrobial Resistance Surveillance Network in veterinary medicine (EARS-Vet) which will register veterinary clinical isolates (Mader et al., 2021). Clinical isolates from livestock are currently not collected by many surveillance systems. The French surveillance network for antimicrobial resistance in bacteria from diseased animals (RESAPATH) in France offers the voluntary submission of 14 clinical isolates (Cazeau et al., 2022). Another large surveillance strategy exists in the US. Data on resistant isolates are collected through the Veterinary Laboratory Investigation and Response Network (Vet-LIRN) in partnership with The National Antimicrobial Resistance Monitoring System (NARMS) (U.S. Food and Drug Administration, 2022a; U.S. Food and Drug Administration, 2022b). NARMS monitors and publishes reports of AMR data from enteric isolates from retail meats, food animals, ill people, and companion animals.

#### 3.6.4 Surveillance on a small scale

There were only three surveillance strategies that were considered small-scale. Two interventions were conducted in LMICs (Indonesia and Vietnam) (Yusuf et al., 2018; Ha et al., 2021) and one at a large veterinary teaching hospital in the USA (Ohio) (Feyes et al., 2021). Of the two studies in LMICs, the first study investigated AMU using sales data of veterinary antimicrobials from drug stores in Indonesia (Ha et al., 2021). An app was created for pharmacists to report sales data allowing them to monitor sales and making data available to monitor on a larger scale. The study acknowledges multiple limitations. Three weeks of sales data was extrapolated to 1 year and as such did not account for variations throughout the year. Furthermore, sales do not equate to usage, and no feed additives were accounted for (Ha et al., 2021). The second surveillance strategy measuring AMU was a self-reporting system for farmers in Vietnam (Yusuf et al., 2018). It involved reporting medical usage and disease via a tablet. This offered farmers a way to track their AMU, without a national system. The study followed farmers on the main island for 2 years, excluding rural settings (Yusuf et al., 2018). Two limitations were observed in this study; there was room for reporting inaccuracies and compliance levels were not reported.

#### 3.6.5 Summary of surveillance strategies

Most surveillance strategies within the scope of this review were based in high income countries suggesting there is little data from LMICs – most likely due to the requirements for financial investment and infrastructure. Within the surveillance strategies that do exist, mandatory reporting at the national level appears widespread which helps ensure that isolates received reflect AMR distribution in each setting. However, reporting especially of clinical isolates is voluntary within systems (Cazeau et al., 2022) which risks a fractured picture of the clinical isolate presence and distribution. Another aspect of surveillance that operates with a margin of error is using sales data as a measure of AMU, as it does not account for off-label use and unused medication. Few surveillance interventions that are considered small-scale were reported, and with varying limitations.

### 3.7 Other interventions

The ‘other’ category of interventions (n = 6) included tools provided by the quadripartite to help countries with surveillance systems or self-assessment of their AMR situation (5/6) and one small-scale intervention that focused on policy related to reducing AMU by farmers and AHPs in the United Kingdom. Two examples of the tools from the quadripartite are the FAO Assessment Tool for Laboratories and AMR Surveillance Systems (FAO-ATLASS) (Food and Agriculture Organization of the United Nations, 2020) and WHO Tool for a Situation Analysis of AMR risks in the Food and Agriculture Sectors on a national level (Food and Agriculture Organisation of the United Nations, n.d).

Using the FAO Assessment Tool, either in surveillance mode or laboratory mode, a baseline level of the country’s setup can be assessed, steps for specific improvement identified, and progress made monitorable (Food and Agriculture Organization of the United Nations, 2020). There is limited information on the related impact or outcomes at national levels. The WHO Tool creates a national report on AMR risk and improvements aiming to provide a picture of the current situation and guide decisions based on One Health principles (Food and Agriculture Organisation of the United Nations, n.d). There may be a gap between receiving a report and actioning change. These tools do not have a primary effect on the target population but generally offer guidance to a country’s AMR plan.

## 4 Discussion

This scoping review summarises the existing evidence on interventions focussed on AMR, AMU and AMS in the animal health sector and provides insights into their impact, gaps, and limitations. Interventions targeting AHPs, farmers, and livestock were of interest. The review included 90 studies that reported interventions from around the world, with 19% of those in LMICs. Within the defined primary outcome measurements, there was a broad range of animal sector interventions. The reported interventions mainly focused on changing AMU levels and changing the development and spread of AMR. Within the primary outcome focused on reducing AMU, herd specific interventions with pre and post intervention measurements were common. Interventions aiming to reduce AMR were often experimental with few investigating the environment or end meat levels. There were few interventions focused on changing knowledge and/or attitudes and perceptions. Retention of knowledge and self-reported change was assessed in some of the reported interventions. Interventions involving surveillance were conducted at the national level and reported AMU determined from sales data and AMR based on detection of indicator bacteria.

Although interventions focusing on AMU were reported, it is important to note that a reduction in AMU does not automatically mean a reduction in AMR. Nonetheless, change in AMU is used as a measurement of impact. Evidence on the linkage between a reduction in AMU and AMR is mixed (Bennani et al., 2020). AMU is used, likely because it is more easily quantifiable and the data requires less resource to collect compared to that of resistant isolates (Bennani et al., 2020). The potential mismatch between AMU and AMR should be considered when assessing the impact of an intervention. Ideally, when assessing a reduction in AMU, the presence of AMR should also be measured.

None of the studies that investigated AMU practices assessed the duration of therapy in animals. Although there is a shortage of veterinary data, data in the human medicine sector indicates a change in therapy can have an impact on AMR without affecting treatment efficacy (Llor and Bjerrum, 2014; de Waele and Martin-Loeches, 2018; Spellberg and Rice, 2019). Furthermore, the lack of data on duration of therapy may indicate that a reduction in AMU could be due to fewer animals being treated, or the same number of animals being treated for a shorter period, unless number of animals is accounted for. This aspect requires closer examination in interventions assessing AMU practices.

Interventions assessing changes in development and/or spread of AMR were carried out in both field and experimental conditions in different livestock (cattle, swine, broilers, goats). Although experimental conditions can negate the limitations of field conditions, interventions garnering results in these conditions may not do so in field conditions. For interventions involving experimental conditions, it is important to account for these differences or to follow up the experimental studies with field studies to reflect real-world conditions.

In general, the interventions focussed on change in behaviour, knowledge, and attitude were few or lacking. Aspects on attitude, behaviour, and knowledge were often implicit parts of interventions but not outcome measurements. Interventions that investigated these aspects featured one or two time points or a set knowledge ‘bank’ without determining if the change in knowledge translated into actions or long-term change. Beyond assessing change in knowledge, it is important to investigate if increased knowledge translates to actions that reduce AMR.

In the current review, the animal health interventions in LMICs were scarce; only 19% (16/83) were performed in LMICs. This is a higher percentage compared to the estimate in human medicine of 1 – 2% (Cox et al., 2017). All the animal health related interventions in LMICs were on a small scale (at herd or farm level). The design of the interventions were mostly deemed of low quality, only one study was of high quality. The reasons for the low number of interventions in LMICs are unclear, but it is possible lack of resources is a contributing factor. The quadripartite and other key players at the global level are making efforts to lessen the gap in skills and resources between LMICs and high-income countries. For example, there are several stewardship tools and road maps that were developed and made available to LMICs to facilitate the implementation of AMR policy and interventions at a national level (World Health Organisation, 2016; Seale et al., 2017). Two examples of these are the Wellcome Trust Road Map for LMICs to participate in the global antimicrobial surveillance system (Seale et al., 2017) and the WHO manual for LMICs to implement national action plans to reduce AMR in both human and animal sectors (World Health Organisation, 2016). These tools need to be coupled with research and to focus on barriers while tailoring the AMR interventions to specific country socioeconomic needs and ensuring the output trickles down to farmers and AHPs.

Differences in the target population (farmers, AHPs, etc) and access to antimicrobials were observed in interventions performed in LMICs vs. high-income countries. Within the reviewed studies, more heterogeneity of the target population (farmers, AHP, etc) was found in LMICs. The farmers in high-income countries run mainly large farms. However, in LMICs, there were small-holdings or small-scale farmers (Phu et al., 2021) and communities that keep livestock for their own consumption (Roulette et al., 2017). In general, in high-income countries, the AHPs encompassed licensed and registered veterinarians but in LMICs, this also included unregistered practitioners prescribing antimicrobials. The access to antimicrobials varied across the targeted populations. Access to over-the-counter antimicrobials (Ha et al., 2021), which is not legal in most high-income countries, and through feed mills (Chinwe et al., 2014) exists in LMICs. This contrasts with high-income countries such as in the EU where access to over the counter is restricted and there is a ban on growth promotors (The European Parliament and The Council of the European Union, n.d.). These nuances in the target population and the access to antimicrobials make for a complex environment to promote AMS and to implement related interventions. The above findings suggest there is a need to tailor interventions aimed at restricting access to antimicrobials and promoting AMS to the local LMIC settings and to the targeted population and regulatory environment.

The AMR surveillance strategies were primarily at a national level and were reported mainly in high-income countries. AMR surveillance strategies often involve collection and testing of a range of isolates. The capacity of laboratories, along with infrastructure, technology and human resources can be a major limitation of surveillance activities at a national level. Increasing the capacity and capability of these aspects in LMICs may provide the opportunity for models applied in high-income countries to be used more globally (Fall et al., 2019; Jayatilleke, 2020). Understanding of the current resistance patterns in given settings could help focus and tailor resources to where they are most needed to reduce AMR and to guide the type of interventions needed to change AMR levels.

Cost, or perceived cost of testing and monitoring can serve as a barrier to efforts to tackle AMR. This may be more pronounced for farmers and AHPs who depend largely on livestock for their livelihood (Golding et al., 2019). Only one herd health intervention on Flemish pig farms reported financial related data with respect to interventions, specifically increased profit, and production parameters (Rojo-Gimeno et al., 2016; Postma et al., 2017). Most studies included in this review did not include financial calculations. This information is relevant for farmers, AHPs, and other key players, to assess if given interventions are financially feasible. Demonstrating that an intervention is of value or of benefit in terms of financial gain or improvement of other parameters (e.g., herd health, feed conversion), can facilitate evidence-based decision making and may encourage the uptake and implementation of interventions considered of value by AHPs and farmers. Ensuring financial and practical feasibility in real-world situations and reducing barriers to uptake within AHPs and farming communities are therefore useful targets to consider when exploring strategies to reduce AMR in the animal health sector.

The assessment of AMU in surveillance strategies was mainly performed using sales data. A limitation with use of sales data is that it does not directly translate to usage of antimicrobials. Medicine can be used for a different animal group than it was licensed and sold for. Wastage or unused medicine is not accounted for either. This means the actual usage could be vastly different from the calculated usage. Furthermore, milligram per millilitre (mg/mL) differs between antibiotics resulting in a different number of doses per mL. Some interventions focused on reducing AMU addressed this issue by using dose instead. However, there is no universal way of denoting dose amounts. The European Medicines Agency has defined daily dose based on active substance and administration route based on a mean. Other studies used other dose denominations. These do not account for discrepancies between different drugs and individual doses. Having a universal dose denomination for animal medicines would help make data comparable globally like it is in human medicine (World Health Organization, n.d).

The findings in the current study should be viewed with limitations in mind. General overview searches across multiple sources (databases and websites of organisation) were performed using defined search terms. Even with such a broad search, articles could be missed. To get an increased sensitivity, all the searches could be performed on a country basis. However, this is not feasible within a reasonable time frame considering all the countries at a global level. The quality of the data has not been evaluated in depth. A light touch review of study design has been performed to ensure some level of quality assessment. The quality of some of the literature is limited as some interventions were based on self-reported information. Self-assessment comes with a social desirability bias (Bradburn et al., 2004). For example, participants reporting that they have changed behaviour as the result of an intervention does not necessary mean that this is the case. People tend to over-report “good behaviour” (Bradburn et al., 2004). Understanding the gap between what is reportedly happening and what is happening in relation to change in AMR is critical for generating reliable outcome measurement in AMR interventions.

In addition to self-assessment and social desirability bias, volunteer bias can be a factor in intervention studies. This is especially plausible in those studies assessing knowledge and/or behaviour change with voluntary participation. Many of the studies addressed this limitation but did not correct for it. It is possible that smaller-scale studies with presumed volunteer bias may have had different outcomes than broader mandatory national/regional interventions (Salkind, 2010).

This scoping review only included interventions that reported change in measured outcomes whether successful or not. It is possible additional unsuccessful or even successful interventions were not being published and therefore not accessed or reviewed. There was a range of study designs and types, with some studies performed in field conditions while others were performed in environments created solely for the intervention. This illustrates the importance of understanding and interpreting intervention outcomes within different settings and contexts.

In conclusion, changes in AMU practices of AHPs and farmers and changes in the development and/or spread of AMR were the most frequent primary outcomes measured in the reviewed studies. Change in uptake and use of AMS, along with change in attitude and knowledge changes were measured less. Small-scale and national-level interventions were more common compared to continental or international interventions. Most interventions were performed in field conditions while some AMR interventions were conducted in experimental conditions. Only 19% of interventions took place in LMICs and were conducted primarily on a small scale. Analysis of the financial aspect of interventions was limited along with an understanding of compliance levels. Self-assessment to measure impact was commonly performed which increases the risk of volunteer bias.

Going forward, a focus on implementing and evaluating interventions in LMICs is warranted to ensure that this underrepresented group is included in the international conversation on AMR. Robust interventions that include objective outcome measures (e.g., measurable outcomes vs. self-reporting) both in LMICs and around the world can increase the understanding of the true impact of AMR interventions. Studies that investigate the benefits and financial implications of interventions are necessary to inform feasibility and the impact of interventions and to encourage uptake of AMR interventions by animal health professionals and farmers.

## Author contributions

Conceptualisation, methodology, analysis, and editing: AJ and AE. Data collection, data extraction, synthesis, result reporting, and writing: AJ and AE. Supervision and reviewing: AE and JO. Methodological analysis of intervention studies: AJ and JO. All authors have read and agreed to the submitted version of the manuscript.
