# Supplementary material for: Antimicrobial resistance interventions in the animal sector: scoping review
Source: Front Antibiot. 2023 Aug 31;2:1233698. doi: 10.3389/frabi.2023.1233698 (PMC11732036; doi:10.3389/frabi.2023.1233698)
Supplement: Supplementary file 1 [file Table_1.docx]

Supplementary Material

**Antimicrobial Resistance Interventions in the Animal Sector: Scoping Review**

**Alice B.J.E. Jacobsen, Jane Ogden, Abel B. Ekiri^*^**

*** Correspondence: Abel B. Ekiri** [**ab.ekiri@surrey.ac.uk**](mailto:ab.ekiri@surrey.ac.uk)

**Appendix 1:**

**Search Strategy**

| **Keywords Used** | **Scopus** | **PubMed** | **Web of Science** |
| --- | --- | --- | --- |
| antibiotic AND intervention AND veterinarian | 96 | 252 | 73 |
| antibiotic AND (OR) intervention AND farmer | 118 | 123 | 104 |
| antibiotic AND intervention AND animal | 2094 | 129 | 813 |
| antibiotic AND intervention AND environment | 139 | 28 | 142 |
| antibiotic AND intervention AND Para-veterinarian | 2 | 1 | 2 |
| antimicrobial AND intervention AND animal | 1167 | 147 | 477 |
| antimicrobial AND intervention AND environment | 422 | 136 | 345 |
| antimicrobial AND intervention AND para-veterinarian | 2 | 1 | 2 |
| antimicrobial AND intervention AND veterinarian | 88 | 9 | 82 |
| antimicrobial AND intervention AND farmer | 108 | 10 | 121 |
| antimicrobial AND intervention AND Africa | 178 | 62 | 182 |
| antimicrobial AND intervention AND Asia | 101 | 35 | 101 |
| antimicrobial AND intervention AND south AND america | 21 | 9 | 17 |
| antimicrobial AND intervention AND north AND america | 34 | 14 | 40 |
| antimicrobial AND intervention AND australia | 141 | 44 | 115 |
| antimicrobial AND intervention AND europe | 135 | 29 | 143 |
| bacteria AND intervention AND veterinarian | 27 | 1 | 17 |
| bacteria AND intervention AND farmer | 71 | 8 | 45 |
| surveillance AND veterinary AND antimicrobial | 609 | 283 | 374 |
