# Supplementary material for: Antimicrobial resistance interventions in the animal sector: scoping review
Source: Front Antibiot. 2023 Aug 31;2:1233698. doi: 10.3389/frabi.2023.1233698 (PMC11732036; doi:10.3389/frabi.2023.1233698)
Supplement: Supplementary file 2 [file Table_2.docx]

Supplementary Material

**Antimicrobial Resistance Interventions in the Animal Sector: Scoping Review**

**Alice B.J.E. Jacobsen, Jane Ogden, Abel B. Ekiri^*^**

*** Correspondence: Abel B. Ekiri** [**ab.ekiri@surrey.ac.uk**](mailto:ab.ekiri@surrey.ac.uk)

**Appendix 2:**

**Focus of intervention groups and measures of interest (incl. target population)**

| **Intervention focus** | **Primary measure of interest** | **Examples of secondary measures of interest** | **Target population** |
| --- | --- | --- | --- |
| Change in antimicrobial use practices of animal health professionals (primary focus on change in AMU) *[Outcome – improved antimicrobial use practices/reduced AMU]* | Change in AMU levels | Reduction of volume/weight of AMU on herd level | AHPs, farmers |
|  |  | Reduction of volume of AMU for a specific diagnosis |  |
|  |  | Reduction of volume of AMU for livestock animals on regional, national, or continental level |  |
|  |  | Reduction in volume of use of Critically Important Antimicrobials for Human Medicine |  |
|  |  | Other |  |
|  | Other measures |  |  |
| Change in uptake and use of antimicrobial stewardship by animal health professionals *[Outcome – increased uptake of antimicrobial stewardship]* | Behavioural change | Change in prescribing habits (define the prescribing habits for which change will be measured) | AHPs, farmers |
|  |  | Increased adherence to guidelines |  |
|  |  | Increase in frequency of use of diagnostics e.g., sensitivity testing |  |
|  |  | Using shortest effective duration of therapy |  |
|  |  | Other |  |
|  | Other measures |  |  |
| Change in development and/or spread/distribution of AMR [*Outcome – reduced development and spread of AMR]* | Change in microbiological parameters (change in resistance of microorganisms, change in distribution of resistant microorganisms) | Reduced frequency of bacterial strain | Livestock |
|  |  | Reduced frequency of resistance genes within bacterial strain |  |
|  |  | Reduced frequency of resistance genes detected/isolated in livestock spp at regional, national, or continental level |  |
|  |  | Reduced frequency of resistance genes detected/isolated within herd/environment around herd |  |
|  |  | Reduced frequency of resistance genes detected/isolated in food products (meat, milk, egg, etc) |  |
|  |  | Reduced frequency of detection of resistant microorganisms from (a) animal samples submitted for diagnostic purposes – animal level, or (b) surveillance animal samples tested & reported at lab level, or (c) environmental samples, or (d) animal derived food products |  |
|  |  | Reduced clinical diagnosis of infections caused by resistant microorganisms |  |
|  |  | Other |  |
|  | Other measures |  |  |
| Change in knowledge of appropriate antimicrobial use practices, AMR, and antimicrobial stewardship *[Outcome – improved knowledge of appropriate antimicrobial use practices, AMR, and antimicrobial stewardship]* | Change in knowledge level | Change in knowledge of appropriate antimicrobial use practices | AHPs, farmers |
|  |  | Change in knowledge of AMR (e.g., increased understanding of AMR (microbiological, public health), how AMR spreads and what it effects, the role of farmers/animal health professions in reduction of AMR |  |
|  |  | Knowledge of antimicrobial stewardship |  |
|  |  | Other |  |
| Change in attitudes and perceptions with respect to antimicrobial use, AMR, and antimicrobial stewardship [*Outcome – positive change in attitudes and perceptions to antimicrobial use, AMR, and antimicrobial stewardship]* | Change in attitudes (mindset, feeling) and/or perceptions (view, opinion) | Changes in attitudes and/or perception on AMR | AHPs, farmers |
|  |  | Changes in attitudes and/or perception on importance of AMR reduction |  |
|  |  | Changes in attitudes and/or perception on farmers/animal health profession’s role in reduction of AMR |  |
|  |  | Other |  |
| Surveillance strategies |  | Surveillance of AMU/AM sales | AHP, farmers, Livestock |
|  |  | Surveillance of AMR |  |
| Other |  |  |  |
